# Recent secondary contact, genome-wide admixture, and asymmetric introgression of neo-sex chromosomes between two Pacific island bird species

**DOI:** 10.1371/journal.pgen.1011360

**Published:** 2024-08-22

**Authors:** Elsie H. Shogren, Jason M. Sardell, Christina A. Muirhead, Emiliano Martí, Elizabeth A. Cooper, Robert G. Moyle, Daven C. Presgraves, J. Albert C. Uy

**Affiliations:** 1 Department of Biology, University of Rochester, Rochester, New York, United States of America; 2 PrecisionLife Ltd, Hanborough Business Park, Long Hanborough, Witney, Oxon, United Kingdom; 3 The Ronin Institute, Montclair, New Jersey, United States of America; 4 Department of Bioinformatics & Genomics, University of North Carolina, Charlotte, North Carolina, United States of America; 5 Department of Ecology and Evolutionary Biology, University of Kansas, Lawrence, Kansas, United States of America; University of Wisconsin–Madison, UNITED STATES OF AMERICA

## Abstract

Secondary contact between closely related taxa represents a “moment of truth” for speciation—an opportunity to test the efficacy of reproductive isolation that evolved in allopatry and to identify the genetic, behavioral, and/or ecological barriers that separate species in sympatry. Sex chromosomes are known to rapidly accumulate differences between species, an effect that may be exacerbated for neo-sex chromosomes that are transitioning from autosomal to sex-specific inheritance. Here we report that, in the Solomon Islands, two closely related bird species in the honeyeater family—*Myzomela cardinalis* and *Myzomela tristrami*—carry neo-sex chromosomes and have come into recent secondary contact after ~1.1 my of geographic isolation. Hybrids of the two species were first observed in sympatry ~100 years ago. To determine the genetic consequences of hybridization, we use population genomic analyses of individuals sampled in allopatry and in sympatry to characterize gene flow in the contact zone. Using genome-wide estimates of diversity, differentiation, and divergence, we find that the degree and direction of introgression varies dramatically across the genome. For sympatric birds, autosomal introgression is bidirectional, with phenotypic hybrids and phenotypic parentals of both species showing admixed ancestry. In other regions of the genome, however, the story is different. While introgression on the Z/neo-Z-linked sequence is limited, introgression of W/neo-W regions and mitochondrial sequence (mtDNA) is highly asymmetric, moving only from the invading *M*. *cardinalis* to the resident *M*. *tristrami*. The recent hybridization between these species has thus enabled gene flow in some genomic regions but the interaction of admixture, asymmetric mate choice, and/or natural selection has led to the variation in the amount and direction of gene flow at sex-linked regions of the genome.

## Author summary

When an invasive species colonizes an island and interacts with a closely related resident species, we are provided with a rare opportunity to study the consequences of interbreeding and the factors that keep species distinct. Regions of the genome that evolve rapidly and/or influence mate choice may be especially likely to act as incidental barriers to gene flow. The red *Myzomela cardinalis*, birds in the honeyeater family, have invaded Makira in the Solomon Islands, coming into contact with the endemic, all black *Myzomela tristrami*. We used population genomic analyses of individuals in geographic isolation, and those in geographic contact, to understand the history of these two species and the consequences of their recent range overlap. We found that sex-specific regions of the genome (*i*.*e*., sex chromosomes) were either limited in their ability to move between species, or only moved in one direction, from the invading *M*. *cardinalis* to the resident *M*. *tristrami*. This work highlights how certain regions of the genome may be especially important in defining species boundaries and the generation and maintenance of biodiversity.

## Introduction

When taxa are geographically isolated, it is difficult to know whether or not they are “good” biological species that are no longer reproductively compatible with each other [1–3]. Secondary geographic contact, therefore, provides a kind of “moment of truth” for speciation—an opportunity to test the efficacy of reproductive isolation in sympatry [1,4]. The presence or absence of interspecific pairings and/or hybrid offspring serve as phenotypic proxies for reproductive isolation, but with genetic data we now know that hybridization between seemingly good biological species is not uncommon (reviewed by [5]). A genic view of speciation allows us to distinguish compatible regions of the genome from those that maintain species divergence [6]. Typically, secondary contact is studied in long-standing hybrid or tension zones [7,8], in which the interaction of gene flow, selection, and recombination occurring over hundreds to thousands of generations allows specific loci to be identified that either move between species or are refractory to introgression due to selection and linkage [9–13]. The consequences of hybridization at the initiation of secondary contact may be transient and difficult to observe. Studying systems in which sympatry is hypothesized to have occurred relatively recently can therefore be especially important to understanding the genetic and/or phenotypic factors which either facilitate or prevent gene flow in the earliest stages of secondary contact [14–16].

Interspecific introgression can occur if traits under sexual or natural selection are globally adaptive, increasing fitness in the genomic background of either species [17,18]. Neutral alleles can introgress due to the demographic dynamics of range expansion precipitating the contact event [19]. However, introgression can be limited for locally adaptive alleles involved in sexual, ecological, and/or intrinsic genetic incompatibilities. Sexual incompatibilities may result from differences in courtship signals or mate preferences [1,20,21]. Ecological incompatibilities can result if hybrids possess intermediate phenotypes poorly suited to either parental habitat [22,23]. Intrinsic genetic incompatibilities can reduce the fertility or viability of hybrids [24–26].

Incompatibilities may be especially likely to arise on sex chromosomes, as these regions of the genome are expected to diverge rapidly. Sex chromosomes have lower effective population sizes compared to autosomes and selection in the heterogametic sex can, under some conditions, lead to “faster-X” (or -Z) evolution [27–30]. Although demography, mating system, and dosage compensation may mediate the strength of faster-X evolution [31], empirical evidence confirms that sex-linked regions show elevated substitution rates and rapid divergence of gene expression for a wide range of taxa [29,32,33]. The rapid evolution on sex chromosomes may explain their disproportionately large role in speciation [34,35]. Species recognition [36] and mating behaviors [37] have been mapped to sex-linked loci, suggesting these regions can be important in maintaining species boundaries via sexual incompatibilities [38]. Sex chromosomes are also known to limit gene flow between taxa through genetic incompatibilities that contribute to Haldane’s rule [34,39] and/or large X/Z effect [34,40–44].

Neo-sex chromosomes—often formed by the fusion of an autosome to an existing sex chromosome—experience a shift to sex-specific transmission, becoming heterogametic in one sex and homogametic in the other [45–48]. The rapid evolutionary transition from autosomal to sex-linked inheritance could enrich neo-sex chromosomes for sexual, ecological, and/or genetic incompatibilities, which reduce gene flow between taxa [49–52]. Indeed, neo-sex chromosomes have been implicated in speciation in plants, insects, and fish [47,49,52]. In birds, karyotype evolution was thought to be largely conservative, with sex chromosomes being syntenic [53,54]. However, neo-sex chromosomes have now been discovered in several bird lineages [45,48,55–61]. Despite their potential importance, few studies have considered the role of neo-sex chromosomes in reproductive isolation in avian taxa [62,63].

**Fig 1:**
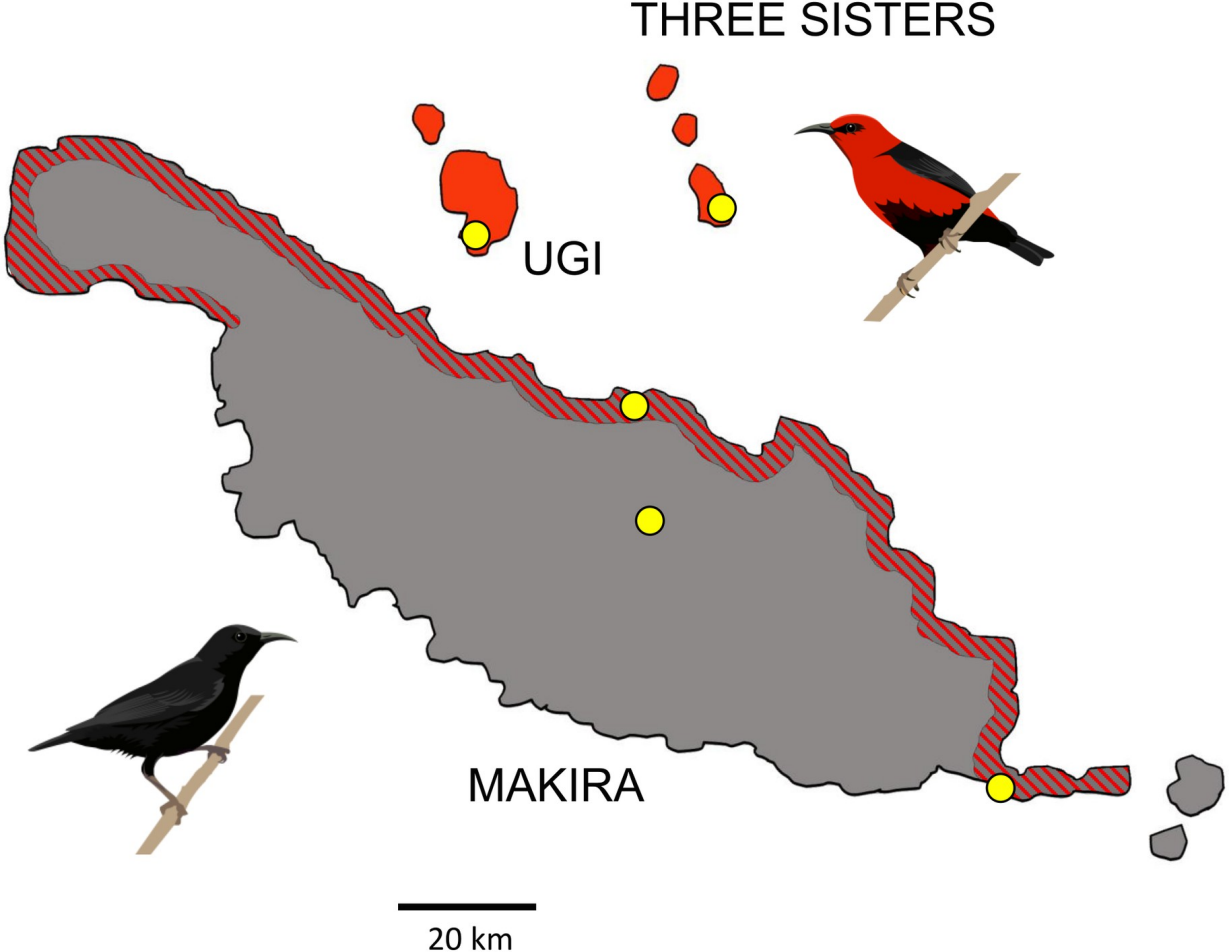
Sampling sites (yellow dots) for allopatric *Mtris* (black bird, gray regions), allopatric *Mcard* (red bird, red regions), and sympatric individuals of both species and phenotypic hybrids (red and gray striped regions). Map downloaded and modified from https://download.geofabrik.de/australia-oceania/solomon-islands.html, illustrations by Emily Powell.

*Myzomela* honeyeaters of the Solomon Islands present an opportunity to study the genomics of reproductive isolation in a system with neo-sex chromosomes [4,64,65]. The sexually monomorphic, all black Sooty honeyeater (*M*. *tristrami*) is endemic to the large island of Makira (Fig 1). The sexually dimorphic, red Cardinal honeyeater (*M*. *cardinalis*), which shared a common ancestor with *M*. *tristrami <*3 mya [66], is distributed across many islands of the South Pacific, including the small satellite islands of Ugi and Three Sisters, 8 km and 20 km from Makira, respectively (Fig 1). Secondary contact occurred recently when *M*. *cardinalis* expanded its range and established a narrow, coastal region of sympatry with *M*. *tristrami*. Birds with intermediate plumage collected on Makira in 1908 were identified as putative hybrids [67] and, in 1927, Ernst Mayr collected phenotypically pure *M*. *cardinalis* as well as putative hybrids as part of the Whitney South Seas Expedition [68]. In subsequent expeditions no phenotypic hybrids were collected, and *M*. *cardinalis* was found to be more abundant than the native *M*. *tristrami* in sympatry [67], leading Mayr and Diamond [4] to propose that hybridization occurred only when conspecific mates were scarce. Preliminary genetic investigations of the system using six nuclear and two mitochondrial loci uncovered evidence for gene flow and for potential neo-sex chromosomes by aligning ddRADseq data to the Zebra Finch reference genome [64,65].

We revisit this classic case of recent secondary contact to address three major questions. (1) What is the history of divergence and secondary contact between *M*. *cardinalis* and *M*. *tristrami*? (2) What is the extent of admixture in the newly established region of sympatry? (3) How does the amount and direction of introgression vary across the genome and, in particular, on sex and neo-sex chromosome sequence? To answer these questions, we use a new, high quality reference genome for *M*. *tristrami*, and whole-genome resequencing for 143 individuals, including samples from both allopatric and sympatric populations of each species. We find evidence for bidirectional introgression on autosomes, limited introgression on the ancestral Z and neo-Z regions, and strong asymmetric introgression of W, neo-W, and mitochondria from *M*. *cardinalis* into *M*. *tristrami*.

## Results and discussion

### Neo-sex chromosomes formed by fusion of sex chromosomes with chromosome 5

**Fig 2:**
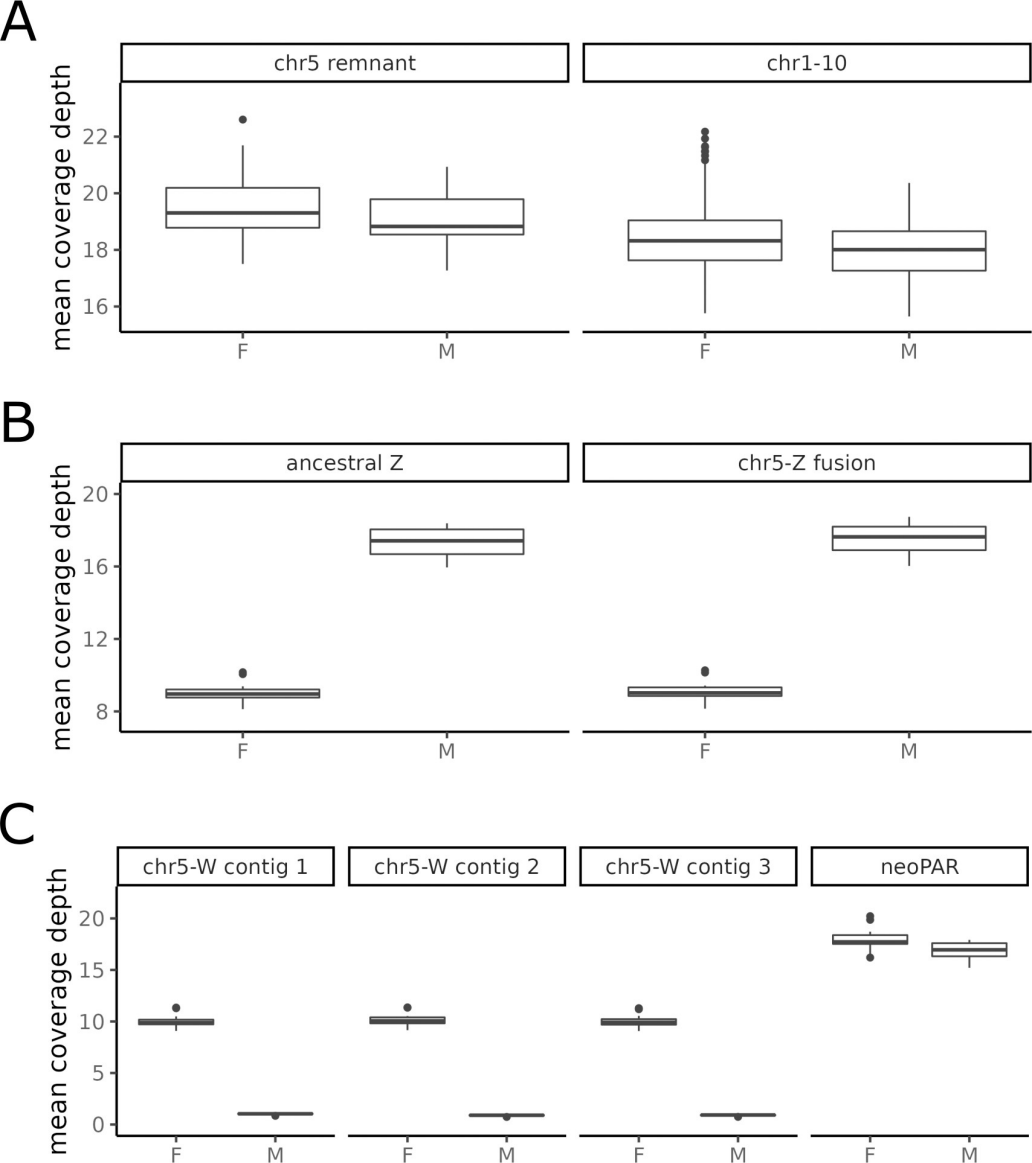
Raw short read sequence coverage from male (M) and female (F) *Mtris* individuals in the allopatric dataset mapped to *Mtris* reference assembly for chromosome 5 remnant contig and representative autosomes (chr1-10; A), Z-linked contigs (B) and W-linked contigs (C).

We generated a highly contiguous, chromosome-level reference assembly for a *Myzomela tristrami* (*Mtris*) female using PacBio HiFi long-read data at approximately 70X autosomal coverage. The primary raw assembly had an N50 of 25.7 Mb and a total length of 1505.7 Mb (S1 Table). After scaffolding and removal of autosomal haplotigs, we conducted a quantitative assessment of conserved avian single-copy orthologs using BUSCO [69], finding an overall BUSCO completeness score of 96.6%. The completed reference genome has a length of 1257.8 Mb and consists of 31 standard autosomes, 11 tentative microchromosomes, a mitochondrial genome, and partially scaffolded Z and W chromosomes. For the Z chromosome we overall find broad collinearity between our assembly and the published assembly of the Z chromosome from a closely related species, *Lichenostomus melanops cassidix* [70], with a major difference: we detect fusion between the sex chromosomes and an autosome (S1 Fig). We find, in particular, that ~86% of chromosome 5 (chr5) is now fused to Z- and W-linked contigs, while the remaining 14% of chr5 (hereafter “chr5 remnant”) assembles separately (S1 Fig). A recently published long-read reference assembly for another honeyeater, *Entomyzon cyanotis*, identified the same fusion between chr5 and ancestral Z sequence and two contigs from an independently assembling region of former chr5 corresponding to our chr5 remnant (their scaffold 13 and contig 14, S1 Fig) [62]. Raw coverage of short-read data from 10 male and 10 female *Mtris* birds (allopatric sample; see below) shows that chr5 remnant has similar coverage in the two sexes, comparable with that of autosomes (Fig 2A). In striking contrast, reads mapping to the chr5 region fused to chromosome Z show a sex difference in mean coverage—~2-fold lower in females than in males—that is inconsistent with being an autosome but consistent with Z-linkage (Fig 2B). We hereafter refer to the Z-fused chr5 region as the neo-Z region. Reads mapping to the chr5 region fused to three of the chromosome W contigs are nearly exclusive to females, inconsistent with being an autosome but consistent with W-linkage (Fig 2C). We refer to this W-fused chr5 region hereafter as neo-W sequence. Although neo-Z and neo-W chr5 derived segments are homologous, our ability to map reads specifically to neo-Z *versus* to neo-W chromosome arms implies appreciable sequence differentiation that could only accrue over time in the absence of recombination. Finally, the remaining ~18Mb of chr5 sequence not captured by either the chr5 remnant or the rearranged neo-Z or neo-W segments is fused to a small segment of ancestral W sequence in our assembly; in the *E*. *cyanotis* assembly this region is part of the “added-Z” contig 4 (S1 Fig). The region shows comparable coverage in both sexes (Fig 2C). We conjecture that this region may be acting as a pseudo-autosomal region (PAR), mediating meiotic recombination on the fused chromosomes, and refer to it as the neo-PAR. We have kept the sex chromosome contigs partially unscaffolded to minimize assumptions of organization. However, we expect the W chromosome to be a contiguous sequence composed of the four contigs identified, while the Z chromosome is expected to be composed of the ancestral Z, the Z1-Z2 fusion contig, and the neo-PAR portion of the fourth W contig. Further in-depth analyses of the origins, structure, and evolution of the neo-sex chromosomes in *Myzomela* will be discussed elsewhere.

### Allopatric populations show distinct demographic histories

**Fig 3:**
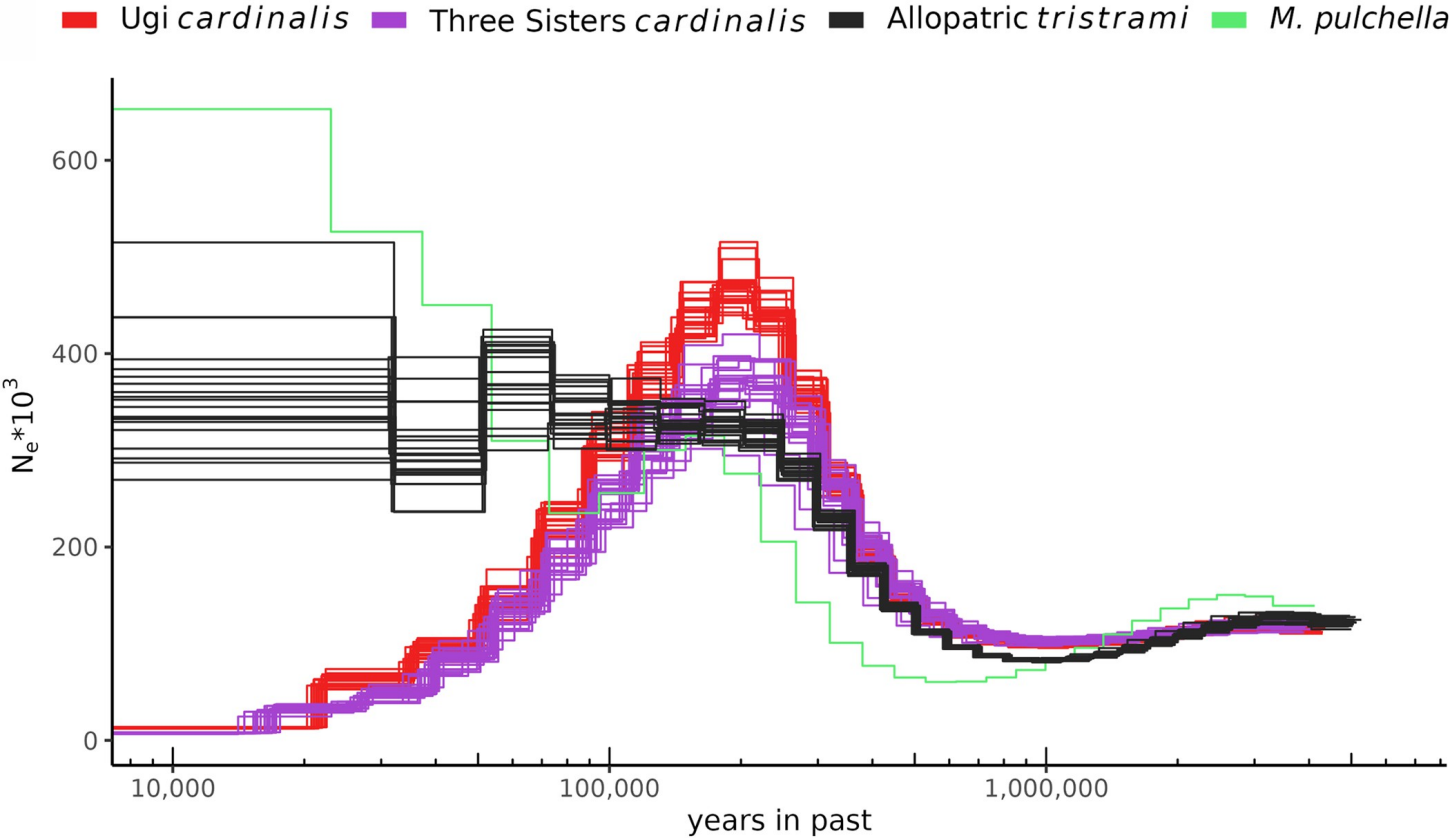
PSMC analyses of allopatric Myzomela. PSMC inferred demographic history using autosomes of allopatric individuals show similar, declining effective populations sizes for *Mcard* on Ugi and Three Sisters, while *Mtris* and the outgroup species *Mpulc* have maintained or increased effective population size in the recent past.

For population genomics analyses, we generated short-read sequence data for a total of 143 individuals: 60 sampled from allopatric and 70 from sympatric regions of the *Mtris* and *M*. *cardinalis* (*Mcard*) ranges; 12 phenotypic hybrids; and 1 individual from an outgroup species, *M*. *pulchella* (*Mpulc*; S2 Table and Fig 1). After alignment to the *Mtris* reference genome and filtering for quality and depth (see Methods), our dataset consisted of 30,283,937 single nucleotide polymorphisms (S3 Table). To infer the speciation and demographic histories of the allopatric populations of *Mtris*, *Mcard* (Ugi and Three Sisters), and the outgroup *Mpulc*, we used pairwise sequential Markov coalescent (PSMC) analyses of the autosomes [71]. These analyses suggest that *Mtris* and *Mcard* diverged ~1.1 mya and, then, both later experienced a period of sustained expansion (Fig 3). During the past ~200Ky, however, the two exhibit starkly different demographic histories: the inferred effective population size (*N*_e_) of *Mtris* has been relatively stable, whereas that for *Mcard* shows evidence of steady decline (Fig 3). The scattered geographic distribution of *Mcard* subspecies across south Pacific islands suggests Ugi and Three Sisters populations may be at the leading edge of the species’ range [4]. We therefore infer that the reduction in *N*_e_ reflects its history of serial founder events during geographic expansion via repeated island colonization.

The smaller *N*_e_ of allopatric *Mcard versus Mtris* is consistent with the lower average nucleotide diversities across all genomic compartments, including autosomes, neo-PAR, Z, neo-Z, W, neo-W, and mitochondria (Tables 1 and S4). Tajima’s *D* for *Mtris* autosomes show an excess of rare SNPs consistent with modest recent population growth, whereas *Mcard* autosomes show an excess of intermediate-frequency SNPs consistent with a recent reduction in *N*_e_ (Table 2). In particular, the Three Sisters population of *Mcard* shows lower nucleotide diversity and more positive Tajima’s *D* values for W and neo-W compared to Ugi *Mcard*, potentially indicating a more recent population reduction and/or founder event; alternatively, the smaller geographic area of Three Sisters (1/6^th^ that of Ugi) may support fewer individuals. Sex chromosome diversity is considerably lower than autosomal diversity in both species. The Z/A ratio of diversity is, for example, much lower than the 0.75 expected on the assumptions of equal sex ratios, random mating, and equal mutation rates in the two sexes: for allopatric *Mtris*, Z/A = 0.646, whereas for Ugi *Mcard*, Z/A = 0.452 and for Three Sisters *Mcard*, Z/A = 0.298 (S5 Table). The lower Z/A ratio in allopatric *Mcard* is also consistent with a population bottleneck in its recent history. The difference between the Ugi *versus* Three Sisters populations of *Mcard* in Z/A nucleotide diversity may reflect population-specific demographic histories and/or sweeps in the Z/neo-Z region [72]. Together, these data suggest that the allopatric *Mcard* populations are relatively new arrivals to Ugi and Three Sisters. Their most recent dispersal event was to Makira, where they encountered the historically stable population of the endemic *Mtris*. A recent invasion of Makira by *Mcard* is consistent with the phenotypic observations of expeditions in the 20^th^ century and the hypothesized history proposed in the literature [4]. We next turn to the genomic consequences of secondary contact.

**Table 1:** Nucleotide diversity, *π*.

population	n	autosome	neo-PAR	Z	neo-Z	W	neo-W	mtDNA
*Myzomela cardinalis*								
Allopatry	40	0.0023 (7 x 10^−6^)	0.0025 (3.8 x 10^−5^)	0.0009 (1.9 x 10^−5^)	0.0008 (2 x 10^−5^)	5 x 10^−6^ (1 x 10^−6^)	4 x 10^−6^ (0)	0.0004 (NA)
Sympatry	40	0.0025 (7 x 10^−6^)	0.0026 (4.1 x 10^−5^)	0.0010 (1.9 x 10^−5^)	0.0007 (2.1 x 10^−5^)	5 x 10^−6^ (1 x 10^−6^)	4 x 10^−6^ (0)	0.0002 (NA)
*Myzomela tristrami*								
Allopatry	20	0.0028 (9 x 10^−6^)	0.0034 (4.2 x 10^−5^)	0.0017 (2 x 10^−5^)	0.0015 (1.7 x 10^−5^)	2.3 x 10^−5^ (2 x 10^−6^)	2 x 10^−5^ (0)	0.0013 (NA)
Sympatry	30	0.0029 (9 x 10^−6^)	0.0034 (4.1 x 10^−5^)	0.0018 (2.1 x 10^−5^)	0.0015 (1.7 x 10^−5^)	0.0006 (1.5 x 10^−5^)	0.0006 (4 x 10^−6^)	0.0138 (NA)

Nucleotide diversity (*π*) averaged across 50 kb windows, standard error in parentheses for each sampled population of phenotypic parental *Myzomela cardinalis* and *M*. *tristrami*. Number of individuals for each species/sampling region shown in column **n**.

**Table 2:** Tajima’s *D*.

population	autosome	neo-PAR	Z	neo-Z	W	neo-W	mtDNA
*Myzomela cardinalis*							
Ugi	0.6466 (0.0059)	0.5342 (0.0336)	0.3706 (0.0402)	0.4694 (0.059)	-0.2637 (0.067)	-0.2402 (0.0263)	-1.4813 (NA)
Three Sisters	0.7613 (0.008)	0.5563 (0.0468)	0.1301 (0.042)	-0.0289 (0.0644)	0.0804 (0.0698)	0.0779 (0.0263)	0.9942 (NA)
Sympatry	0.2976 (0.0056)	0.3412 (0.0366)	-0.3043 (0.0435)	-0.9828 (0.061)	-0.2246 (0.0749)	-0.3755 (0.0246)	-1.3924 (NA)
*Myzomela tristrami*							
Allopatry	-1.1067 (0.0026)	-1.0639 (0.0125)	-0.8908 (0.0094)	-0.7560 (0.0149)	-0.3088 (0.046)	-0.3165 (0.0194)	-0.8481 (NA)
Sympatry	-1.2067 (0.0024)	-1.1609 (0.0118)	-1.0246 (0.0092)	-0.9158 (0.0141)	1.7281 (0.0222)	2.0505 (0.0042)	1.5222 (NA)

Tajima’s *D*, averaged across 50 kb windows, standard error in parentheses for each sampled population of phenotypic parental *Myzomela cardinalis* and *M*. *tristrami*.

### Autosomal loci introgress in both directions at secondary contact

We captured 187 birds in sympatry. Of these, 68 were phenotypically *Mtris*, 107 were phenotypically *Mcard*, and 12 were identified as “phenotypic hybrids”—individuals with plumage characteristics clearly intermediate between *Mtris* and *Mcard* (*e*.*g*., mostly black with some red feathers). These individuals raise the possibility that our sample includes backcross or advanced generation hybrids that are indistinguishable from the parental species. To test for the possibility of cryptic hybrids, we start by focusing on analyses of autosomal regions of the genome. We used five approaches to characterize autosomal admixture. First, simple summaries of differentiation and divergence restricted to phenotypically parental individuals (excluding phenotypic hybrids) support admixture between sympatric populations (Tables 3, S7 and S8). Autosomal differentiation (*F*_ST_) between allopatric *Mcard* and *Mtris* is 0.282, whereas that for sympatric *Mcard* and *Mtris* drops to *F*_ST_ = 0.197 (Table 3). The difference in absolute divergence between species is less dramatic but still larger in allopatry (*d*_xy_ ≈ 0.0035) than in sympatry (*d*_xy_ = 0.0033; S8 Table). The observed shifts in estimated differentiation and divergence in sympatry when looking solely at phenotypically pure *Mcard versus Mtris* are consistent with introgression.

**Table 3:** Population differentiation, *F*_ST_.

population comparison	autosome	neo-PAR	Z	neo-Z	W	neo-W	mtDNA
*Myzomela cardinalis*							
Allopatry vs. Sympatry	0.0323 (2 x 10^−4^)	0.0326 (0.0013)	0.0508 (0.0016)	0.0668 (0.0033)	-0.0067 (0.0061)	-0.0032 (0.002)	0.0019 (NA)
*Myzomela tristrami*							
Allopatry vs. Sympatry	0.0061 (1 x 10^−4^)	0.0047 (2 x 10^−4^)	0.0054 (3 x 10^−4^)	0.0051 (5 x 10^−4^)	0.1548 (8 x 10^−4^)	0.1557 (2 x 10^−4^)	0.1233 (NA)
Heterospecific							
Allo. *Mcard* vs. Allo. *Mtris*	0.2818 (0.001)	0.2757 (0.0061)	0.5921 (0.0048)	0.6606 (0.0052	0.9917 (7 x 10^−4^)	0.9924 (2 x 10^−4^)	0.9831 (NA)
Allo. *Mcard* vs. Sym. *Mtris*	0.2423 (9 x 10^−4^)	0.2426 (0.0054)	0.5704 (0.0048)	0.644 (0.0049)	0.7409 (8 x 10^−4^)	0.7411 (2 x 10^−4^)	0.8017 (NA)
Allo. *Mtris* vs. Sym. *Mcard*	0.2354 (9 x 10^−4^)	0.2509 (0.0066)	0.5717 (0.005)	0.6630 (0.0067)	0.9920 (6 x 10^−4^)	0.9927 (2 x 10^−4^)	0.9855 (NA)
Sym. *Mcard* vs. Sym. *Mtris*	0.1972 (8 x 10^−4^)	0.2178 (0.0059)	0.5511 (0.005)	0.6433 (0.0064)	0.7411 (7 x 10^−4^)	0.7413 (2 x 10^−4^)	0.8041 (NA)

*F*_ST_ averaged across 50kb windows for each region of the genome, for pairwise comparisons of phenotypic parental populations of *Myzomela cardinalis (Mcard)* and *M*. *tristrami (Mtris)*. Allopatry and sympatry abbreviated as Allo. and Sym., respectively. Standard errors in parentheses.

**Fig 4:**
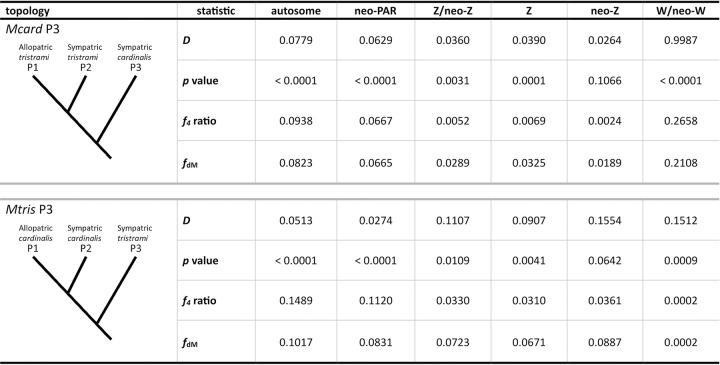
Test of introgression, ABBA-BABA results. A positive *D* statistic indicates gene flow between P2 and P3, and the *p* value specifies whether *D* is significantly different from zero, using block-jackknife procedure. The *f*_*4*_ ratio is calculated by splitting P3 population into two subsets to calculate the admixture proportion, assuming unidirectional introgression P3 → P2. The average *f*_dM_ statistic is calculated using 100 SNP non-overlapping windows (W/neo-W calculated in 50 SNP non-overlapping windows) where *f*_dM_ ≥ 0, indicating either no gene flow (*f*_*dM*_ = 0) or gene flow between P2 and P3 (*f*_*dM*_ > 0). Allopatric *M*. *cardinalis* includes samples from both Ugi and Three Sisters. *M*. *pulchella* is the P4 outgroup in both topologies.

**Fig 5:**
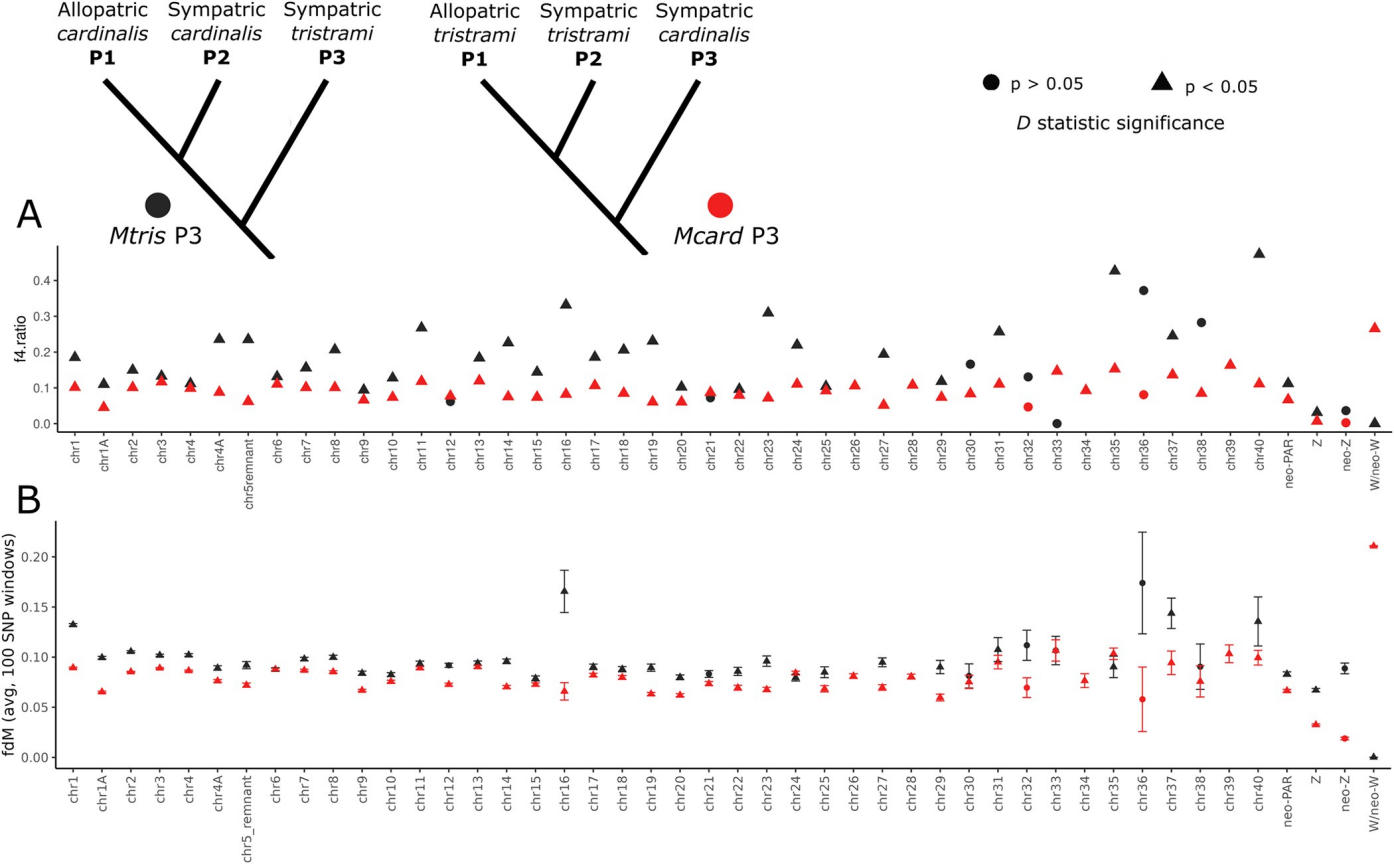
Admixture statistics (*f*_4_, A; *f*_dM_, B) for autosomes and sex chromosome regions. Color of the point indicates which topology the statistic was calculated for (*Mtris* P3 or *Mcard* P3), and shape of the point indicates whether the *D* statistic for that chromosome/region was significantly different from zero, determined using the block-jackknife procedure. *f*_dM_ statistics were calculated across 100 SNP windows, restricted to windows where *f*_dM_ ≥ 0 (potential gene flow between sympatric populations) and averaged, with bars showing standard error across windows for that chromosome/region. Admixture *f*_4_ ratios calculated for *Mtris* P3 taxa on chr 26, 28, 34, and 39 used an alternative topology indicating nonsignificant introgression from sympatric *Mtris* into allopatric *Mcard*, shown in S2 Fig.

Second, to infer the amount and direction of introgression between species we conducted ABBA-BABA analyses using the program Dsuite [73] to calculate the *D-*statistic, *f*_*4*_, and *f*_*dM*_ admixture statistics (Fig 4). ABBA-BABA uses absolute counts of the distribution of derived alleles shared among four taxa using the topology (((P1,P2)P3)P4) to determine if gene flow has occurred among the three ingroup taxa [74]. We used *Mpulc* as the P4 outgroup and analyzed two different topologies: “*Mcard* P3” with allopatric *Mtris* (P1), sympatric *Mtris* (P2), and sympatric *Mcard* (P3) as the ingroup; and *“Mtris* P3” with allopatric *Mcard* (P1), sympatric *Mcard* (P2), and sympatric *Mtris* (P3) as the ingroup. For both topologies, *D* statistics were positive and significant, indicating excess sharing of derived alleles between sympatric *Mcard* and *Mtris* consistent with autosomal gene flow. The *f*_*4*_ statistic calculates a proportion of admixture assuming unidirectional gene flow from P3 into P2. A slightly higher admixture proportion (*f*_*4*_ ratio) calculated for the *Mtris* P3 topology (0.15) suggests more gene flow from sympatric *Mtris* (P3) into sympatric *Mcard* (P2) than the reverse (0.09; Fig 4 and Fig 5A). The *f*_*dM*_ statistic also estimates admixture [75] and does not assume unidirectional gene flow but nevertheless echoes the *f*_*4*_ ratio, showing slightly higher and more variable admixture in the *Mtris* P3 topology (Fig 5B). Parsing signals of introgression by chromosome, *D* statistics are significant for both topologies for most autosomes (74% for *Mtris* P3, 95% for *Mcard* P3; Figs 5 and S2). The relatively lower overall level of *Mcard*→*Mtris* introgression on autosomes (*f*_*4*_ statistic) may be attributable to relative abundance of the two species, to asymmetric mate preferences, and/or to more efficient selection against foreign alleles in the *Mtris* population owing to its larger effective population size.

**Fig 6:**
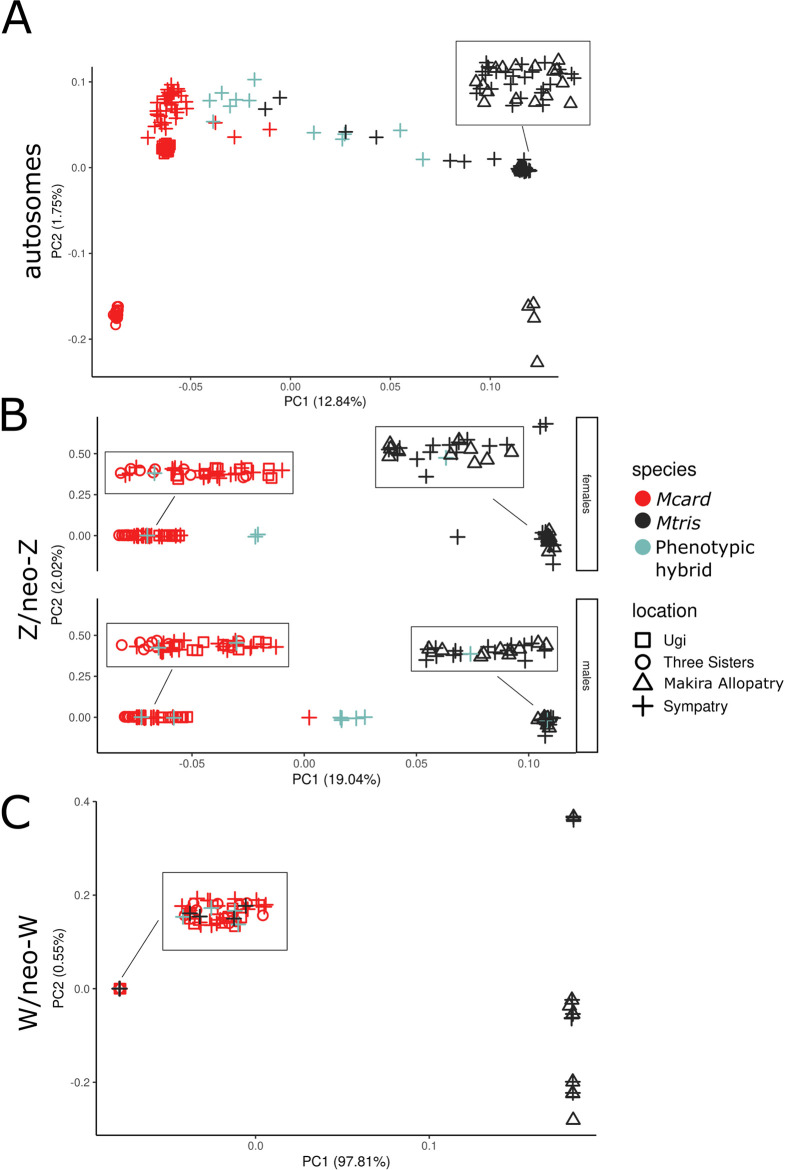
Principal components analysis of autosomal (A), Z/neo-Z (B) and W/neo-W (C) sequence. Symbol color represents phenotypic species assignment while symbol shape indicates sampling locality. Inset boxes show points jittered for visualization. Plot for Z/neo-Z is separated by sex to distinguish homogametic males and heterogametic females.

Third, we used principal component analyses (PCA) to identify admixed individuals. PCA revealed clear separation of allopatric *Mcard* and *Mtris* along principal component 1 (PC1, Fig 6A). As expected, phenotypic hybrids were intermediate in PC1 values (Fig 6A). Notably, several phenotypic *Mcard* and *Mtris* individuals also fell between the two clusters of parental species. These “cryptic hybrids” are likely backcross or advanced generation hybrids.

**Fig 7:**
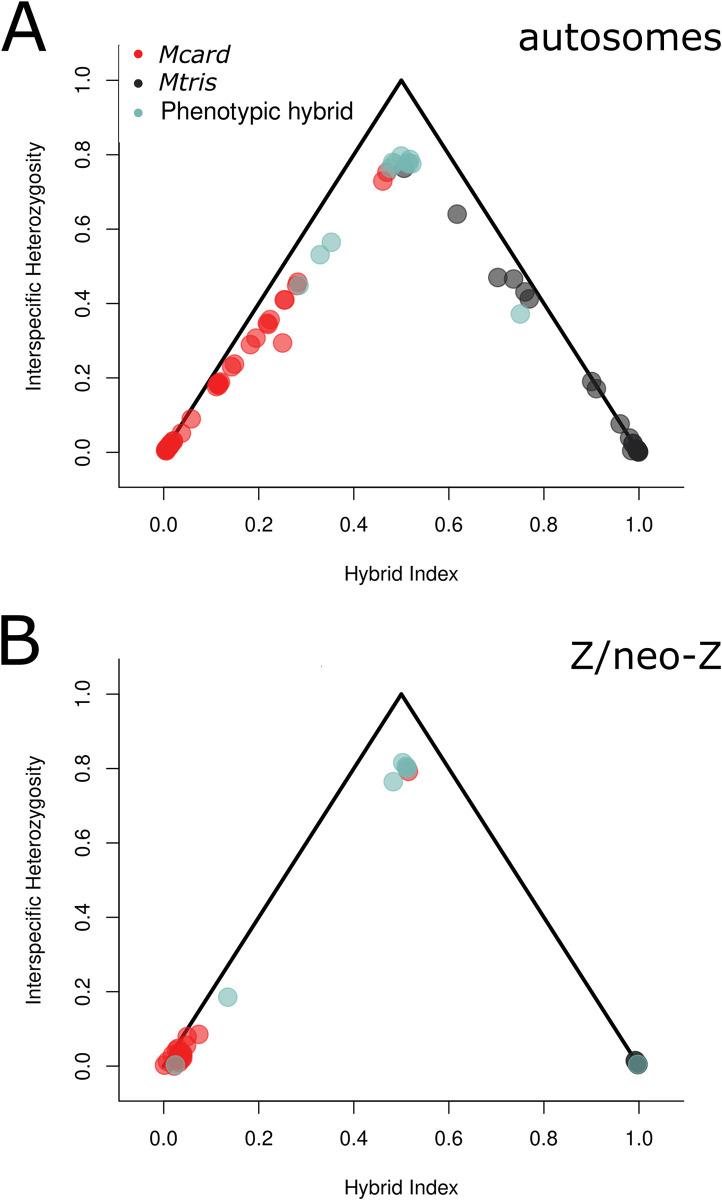
Triangle plots showing the relationship between interspecific heterozygosity and hybrid index calculated using 2,449 autosomal (A) and 37,613 Z/neo-Z (B) SNPs fixed between species in allopatry. Circles are sympatric individuals of both sexes (A) or sympatric males (B), colored by phenotype (see legend). F1 individuals fall at the maximum heterozygosity and hybrid index ≈ 0.5, while advanced generation backcrosses fall along the legs of the triangle as interspecific heterozygosity declines and hybrid index is closer to 0 (*Mcard* ancestry) or increases to 1 (*Mtris* ancestry).

To explicitly test for production of F_1_s and advanced generation backcrosses we used autosomal SNPs fixed between allopatric *Mcard* and *Mtris* to estimate the interspecific heterozygosity and the hybrid index for each sympatric individual (Fig 7A). We see clear evidence of F_1_s with near-maximum interspecific heterozygosity and hybrid indices ≈ 0.5. Most are phenotypic hybrids (*n* = 8), but phenotypic *Mcard* (*n* = 2) and *Mtris* (*n* = 1) also appear to be F_1_ individuals. We detect 28 backcross hybrids in both crossing directions but no F_2_ individuals (Fig 7A). The absence of F_2_s may be due to limited sampling, low frequency of F_1_s limiting opportunities for F_1_ x F_1_ matings, the mating behavior of hybrids, or fitness breakdown in F_2_s.

**Fig 8:**
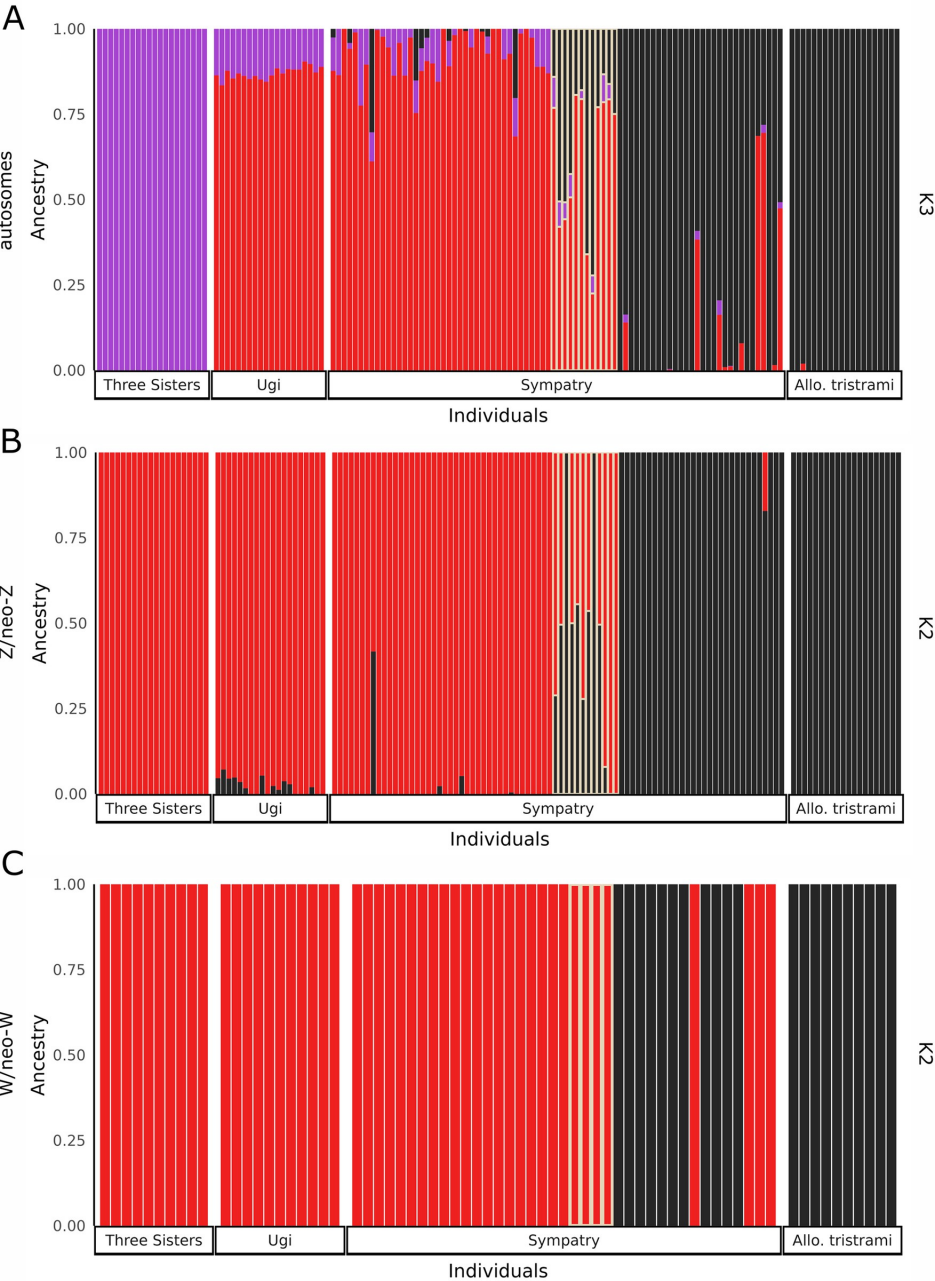
Proportion ancestry calculated in ADMIXTURE for autosomes (A), Z/neo-Z (B), and W/neo-W (C). Autosomes are shown at K = 3 (see S3 Fig for K = 2), while Z/neo-Z and W/neo-W shown at K = 2. Individuals are grouped by sampling location. Phenotypic hybrids in sympatry are outlined in yellow, with phenotypic *Mcard* in sympatry to the left and phenotypic *Mtris* in sympatry to the right of phenotypic hybrids.

Finally, we used ADMIXTURE to estimate individual ancestry proportions [76]. Cross-validation error was minimized when the number of groups (*K*) was equal to two (S3 Fig). However, *K* = 3 was similarly well-supported for autosomal sites and informative about structure between the Ugi and Three Sisters *Mcard* populations (Fig 8A; see below for further discussion of *Mcard* allopatric populations). All phenotypic hybrids, as well as several individuals in sympatry (phenotypically *Mtris* and phenotypically *Mcard*) show autosomal ancestry from both species. In summary, autosomal sequence indicates bidirectional admixture between *Mcard* and *Mtris* in sympatry. The presence of advanced-generation backcrosses in our sample confirms that some F_1_ hybrids are fertile. The relative abundance of *Mcard* in sympatry (107 of 187 individuals captured in 2008–2015) and the continued production of phenotypic hybrids suggests ecological incompatibilities do not strongly influence reproductive isolation in the region of sympatry, although additional work is necessary to confirm this.

### New pseudo-autosomal region shares patterns of introgression with autosomes

The new pseudo-autosomal region, or neo-PAR, is a region of the former autosome, chr5, that is now linked to the neo-sex chromosomes but has continued to recombine in both sexes. The neo-PAR therefore serves as an important point of comparison with the neo-sex chromosome regions that have limited recombination. The neo-PAR has nucleotide diversity similar to autosomes, likely preserved by a history of uninterrupted recombination since the initial formation of the neo-sex chromosome fusion (Table 1). Differentiation within and between species on the neo-PAR is also in line with that of autosomes (Table 3). Finally, signals of introgression on the neo-PAR parallel those of the autosomes, with significant *D* statistics for both topologies. Admixture statistics were only slightly lower for the neo-PAR than for autosomes as a whole, and tracked values of individual autosomes of similar size (Fig 4 and S4 Fig). Thus, despite transitioning from autosomal sequence to a part of the sex chromosomes, the neo-PAR region appears to have a history similar to the autosomes. We now compare the bidirectional pattern of autosomal and neo-PAR gene flow to introgression patterns observed on sex and neo-sex chromosome sequence, which we expect to differ due to variable ploidy and recombination (S8 Table).

### Z /neo-Z refractory to introgression

The Z/neo-Z linked sequence shows evidence of admixture, but the degree of introgression is markedly reduced compared to autosomes. Differentiation on the Z and neo-Z regions between *Mcard* and *Mtris* is slightly lower in sympatry (Z: *F*_ST_ = 0.551, neo-Z: *F*_ST_ = 0.643) than in allopatry (Z: *F*_ST_ = 0.592, neo-Z: *F*_ST_ = 0.661), but is overall much higher than autosomes (Table 3). Divergence between *Mcard* and *Mtris* in allopatry is similar to that in sympatry (S8 Table). In the ABBA-BABA analysis, *D* statistics for the Z/neo-Z region were significant, consistent with gene flow between sympatric *Mcard* and *Mtris*, but *f*_*4*_ and *f*_*dM*_ admixture statistics were lower than those estimated for autosomal sites (Fig 4 and Fig 5). We also estimated admixture separately for ancestral Z and neo-Z regions of the Z chromosome. Admixture statistics (*D*, *f*_*4*_ and *f*_*dM*_) for the ancestral Z are consistent with those estimated from the combined Z/neo-Z region (Fig 4). However, the neo-Z region yielded more complex results. First, *D* statistics for the neo-Z are not statistically significant in either the *Mcard* P3 or the *Mtris* P3 topologies (Fig 4), consistent with limited or no introgression. The *Mcard* P3 *f*_4_ and *f*_*dM*_ admixture statistics for neo-Z sites are lower than those for the autosomes and the Z, suggesting *Mcard*→*Mtris* neo-Z introgression may be negligible. For *Mtris* P3, however, interpretation is more difficult: *f*_4_ admixture ratios are similar between Z and neo-Z while *f*_*dM*_ statistics were on average slightly higher in neo-Z than Z. However, we note again that *D* statistics for the neo-Z region were non-significant and admixture statistics should therefore be interpreted with caution.

Analyses of individual genotypes revealed F_1_, backcross, and advanced generation backcross individuals in our sample. For PCA of the Z/neo-Z, we separated homogametic males and heterogametic females for visualization (Fig 6B). *Mcard* and *Mtris* clearly separated along PC1. Five of eight phenotypic hybrid males and one phenotypic *Mcard* male were intermediate in PC1, potentially F_1_ individuals heterozygous for species’ Z/neo-Z haplotypes. However, there were also phenotypic hybrid males which fell within the species’ clusters at either end of PC1, indicating those individuals may be advanced generation backcross hybrids homozygous for Z/neo-Z haplotypes from either *Mcard* (*n* = 2) or *Mtris* (*n* = 1). Although females only have one Z haplotype, three individuals had intermediate values of PC1 (Fig 6B). These females are likely backcross hybrids carrying recombinant Z/neo-Z chromosomes with both *Mcard* and *Mtris* sequence. Plotting hybrid index against interspecific heterozygosity for diploid males confirmed five of eight phenotypic hybrid males and one phenotypic *Mcard* male were heterozygous for their Z/neo-Z haplotype (Fig 7B). Individual-level estimates of ancestry proportions calculated in ADMIXTURE showed some but not all phenotypic hybrids were admixed for their Z/neo-Z haplotype, while only two phenotypically parental individuals had appreciable ancestry from the other species (Fig 8B).

Together, these results show introgression of Z/neo-Z in sympatry is limited relative to autosomal sequence. Aggregated, population-level estimates of differentiation are similar in allopatric and sympatric species comparisons, while estimates of introgression and admixture proportion are very low, and in the case of the neo-Z, not significant. Although sympatric individuals span a range of autosomal admixture, only a few carry Z/neo-Z genetic material from both species (S5A Fig). The absence of sex chromosome recombination in female hybrids limits production of individuals with admixed ancestry on the Z/neo-Z chromosome, in contrast to autosomes which assort independently and have the potential to recombine in both male and female hybrids. However, admixture statistics for Z and neo-Z are lower than those calculated for single autosomes of similar size and nucleotide diversity (S4 and S6 Figs). Thus, it seems likely that selection, perhaps in combination with reduced recombination, limits introgression of Z/neo-Z.

### Strong, asymmetric introgression of W/neo-W and mitochondria

The W, neo-W, and mitochondrial sequence are all maternally co-transmitted to females, while males receive only mitochondrial sequence from their mother. *Mcard* and *Mtris* carry distinct sets of W, neo-W, and mitochondrial haplotypes and in our sampling of sympatric individuals all three regions show strong, asymmetric introgression from *Mcard* into *Mtris*. Interspecific differentiation between *Mtris* and *Mcard* is lower in sympatry (*F*_ST_ = 0.74) than in allopatry for W and neo-W regions (*F*_ST_ = 0.99; Table 3), consistent with introgression. As a result, *Mtris* now shows within-species differentiation between allopatric and sympatric individuals at W/neo-W (*F*_ST_ = 0.15) and mitochondrial sites (*F*_ST_ = 0.12) but not autosomal ones (*F*_ST_ < 0.01; Table 3). The introgression of *Mcard* W/neo-W and mitochondrial haplotypes has produced a striking positive shift in Tajima’s *D* for sympatric *Mtris* birds (Table 2). ABBA-BABA analyses for W/neo-W haplotypes further support asymmetric introgression from *Mcard* into *Mtris*. The *Mcard* P3 topology showed a significant *D* statistic and an *f*_4_ admixture proportion of 0.27; *Mtris* P3 topology also had a significant *D* statistic, but the *f*_4_ admixture proportion indicating gene flow *Mtris → Mcard* was ≈ 0 (Fig 4 and Fig 5). The extreme W/neo-W introgression from *Mcard* into *Mtris* is particularly apparent when plotting *Mcard* P3 *f*_dM_ values against chromosome/region nucleotide diversity calculated in the allopatric *Mtris* population (S6 Fig). For other genomic regions a slightly positive relationship between nucleotide diversity and introgression is expected and observed, but despite negligible nucleotide diversity on W/neo-W, we observe strong introgression indicated by a high *f*_dM_ statistic.

Movement of *Mcard* W/neo-W haplotypes into *Mtris* is also evident in analysis of individual genotypes. All phenotypic hybrid females and four of 15 phenotypic *Mtris* females cluster with *Mcard* individuals in PCA of W/neo-W sites, whereas none of the sympatric *Mcard* females cluster with *Mtris* (Fig 6C). Females carrying the *Mcard* W/neo-W haplotype also exhibit a wide range of autosomal ancestry, while those with *Mtris* W/neo-W are exclusively *Mtris* in autosomal background (S5B Fig). All sympatric *Mcard*, phenotypic hybrid, and four of 15 sympatric *Mtris* females have only *Mcard* ancestry in ADMIXTURE analyses (Fig 8C). Reassuringly, the proportion of *Mtris* females carrying W/neo-W haplotypes (4/15 = 0.267) matches the *Mcard→Mtris f*_4_ admixture ratio calculated using the *Mcard* P3 topology (Fig 4). The haplotype network for the full mitochondrial genome shows the same asymmetric pattern of introgression and provides insight to the maternal contribution to hybrid males. Mitochondrial haplotypes for *Mtris* are restricted to phenotypic *Mtris*, whereas *Mcard* mitochondrial haplotypes are carried by phenotypic hybrids and by sympatric *Mcard* and *Mtris* (S7 Fig). These results extend and confirm previous analyses of mitochondrial markers [65]. Consistent with asymmetric introgression, our sample has four phenotypic hybrid females, four phenotypic *Mtris* females, and 12 admixed males carrying *Mcard* W/neo-W and/or mitochondrial haplotypes. These individuals are therefore ultimately the product of crosses between *Mcard* females and *Mtris* males; we observe no admixed individuals produced by the reciprocal cross (*i*.*e*., carrying *Mtris* W/neo-W and/or mitochondrial haplotypes). Notably, the *Mcard→Mtris* introgression we observe for W/neo-W and mtDNA is counter to the expected direction of local→invading species resulting from initial introgression of local alleles and subsequent dilution of invading alleles [19,77]. The absence of admixed birds with *Mtris* W/neo-W or mtDNA in our dataset may reflect our limited sample size and/or limited rate of successful hybridization involving *Mtris* females. However, even with modest sample sizes we find that W/neo-W/mitochondrial introgression is significantly asymmetric: *Mtris* individuals are more likely than *Mcard* individuals to carry heterospecific W/neo-W (Fisher’s exact test, *p* = 0.026, females only, S9 Table) and/or mitochondria (Fisher’s exact test, *p* = 0.005, males and females, S9 Table).

We propose three potential explanations for asymmetric introgression of the *Mcard* W/neo-W and mitochondria. First, if matings between *Mtris* females and *Mcard* males occur, Haldane’s rule for lethality predicts a dearth of F_1_ hybrid daughters [39], which is often manifest in only one direction of the species cross due to genetic incompatibilities involving sex chromosomes or mitochondria [25,78,79]. There are, however, several reasons to consider alternative explanations. For one, Haldane’s rule for hybrid sterility in both directions tends to evolve before hybrid lethality [80]. In addition, hybrid lethality in birds tends to occur between much older species pairs [3,81]. Because mitochondrial sequence is co-inherited with W/neo-W and shares the pattern of asymmetric introgression, mitonuclear interactions or incompatibilities may also or instead be the target of selection [82,83]. It remains possible, however, that rapid evolution of the neo-W has accelerated the evolution of hybrid lethality or mitonuclear incompatibilities [50].

Sexual incompatibilities provide a second explanation for asymmetric introgression. Asymmetry in mate choice or mate availability may limit crosses between *Mtris* females and *Mcard* males [38]. Indeed, Mayr and Diamond [4] proposed that hybridization in sympatry initially occurred due to a lack of conspecific mates for recently arrived *Mcard*. Since then, however, the relative abundance of *Mcard* has surpassed that of the endemic *Mtris*. It therefore seems unlikely that *Mcard* females are constrained to pair with heterospecific males [65]. In fact, for a sample of molecularly sexed individuals captured foraging at flowering trees, sympatric *Mcard* shows a ratio of 1.1 males per female (*n* = 81), whereas *Mtris* has an even higher ratio of 2.1 males per female (*n* = 56; S10 Table). Nevertheless, phenotypic hybrids and admixed individuals continue to be observed decades after invasion. It is therefore possible that introgression of maternally coinherited sequences from *Mcard* into *Mtris* genomic backgrounds occurs via asymmetric mate choice or natural selection [84,85].

Positive selection which favors *Mcard* W/neo-W and/or mitochondria in the *Mtris* population is a third potential explanation for the asymmetric introgression we observe. The *Mcard* W/neo-W/mitochondria could, for instance: carry some globally adaptive mutation(s) absent from *Mtris* [86]; have a smaller load of unconditionally deleterious mutations [87]; and/or possess a transmission advantage (meiotic drive) in an *Mtris* genetic background [88]. Future work is necessary to distinguish among the three possible drivers of W/neo-W and mitochondrial introgression from *Mcard* into *Mtris*.

### More than one source for sympatric *Mcard* population

To determine if the *Mcard* population(s) that invaded Makira originated from Ugi, from Three Sisters, or from both, we assessed relationships between the two allopatric *Mcard* and the sympatric *Mcard* populations. We identified very few alleles private to sympatric *Mcard* or fixed between sympatric *Mcard* and either allopatric *Mcard* population, while many alleles were shared among all three *Mcard* populations (S11 and S12 Tables). Three Sisters *Mcard* show distinct ancestry from Ugi *Mcard* in ADMIXTURE analyses allowing for three ancestral groups (K = 3; Fig 8A). Across all genomic sites, differentiation between Ugi and Three Sisters populations of *Mcard* is higher than that between either allopatric population and sympatric *Mcard* (S6 Table). Ugi *Mcard* does appear to have been a stronger contributor to the sympatric *Mcard* population, based on differentiation between the populations and clustering in autosomal PCA (S6 Table and Fig 6A). The mitochondrial network, however, shows that haplotypes otherwise unique to Ugi or to Three Sisters are both present in sympatric *Mcard*, sympatric *Mtris*, and phenotypic hybrids (S7 Fig). Thus, it is likely both Ugi and Three Sisters individuals contributed to the founding population of *Mcard* on Makira.

## Conclusions

Our population genomic study of *Myzomela* honeyeaters in the Solomon Islands sheds light on a system with complex and ongoing introgression following very recent secondary contact of closely related taxa carrying neo-sex chromosomes. Sex chromosomes are known to play a large role in speciation [34]. Neo-sex chromosomes transitioning from autosomal to sex-specific inheritance undergo rapid structural and molecular evolution, which may incidentally contribute to incompatibilities between species [50,51,89]. Consistent with genetic and/or sexual incompatibilities accumulating on sex and neo-sex chromosomes, we see limited introgression of Z/neo-Z. Modest autosomal introgression into both species occurs. Surprisingly, and in contrast to other chromosomes, we observe asymmetric introgression of the W/neo-W and mitochondria. The patterns of introgression that we see highlight the potential for gene flow to vary across the genome, and the importance of sex chromosomes in maintaining species boundaries. Importantly, gene flow is reduced for Z/neo-Z sequence relative to autosomes, possibly owing to its faster accumulation of locally adaptive and/or incompatible substitutions. In contrast, the asymmetric introgression of the W/neo-W suggests either asymmetric mating success (*e*.*g*., an excess of hybrid crosses involving *Mcard* females), mitonuclear or W/neo-W linked incompatibilities which limit introduction of *Mtris* W/neo-W and/or mtDNA into *Mcard* genomic backgrounds, or some form of selection that favors *Mcard* W/neo-W and/or mitochondrial haplotypes in *Mtris*. Further work is of course needed to determine the specific behaviors and/or loci that underlie these patterns. By revisiting the *Myzomela* honeyeater system in Makira and testing predictions of the allopatric model of speciation with genomic data, we build on the work started by Mayr and Diamond [4], providing genomic insights into the “moment of truth” for speciation.

## Materials and methods

### Ethics statement

Sampling methodology was approved by the Institutional Animal Care and Use Committees at the University of Miami (Protocol 12–100) and University of Kansas (Protocol AUS 174–01).

### Samples and sequencing

Between 2008 and 2015 we sampled 40 allopatric *Myzomela cardinalis* from two island groups adjacent to the region of sympatry on Makira (*i*.*e*., Ugi and Three Sisters) and 20 allopatric *M*. *tristrami* from high elevation regions on the island of Makira (Fig 1). We also sampled 82 birds from the low elevation region of sympatry on Makira. Of these, 40 were identified as *M*. *cardinalis*, 30 were identified as *M*. *tristrami*, and 12 were identified as hybrids, based on a phenotype of mostly black plumage with patches of red feathers. In addition, we used a sample of *Myzomela pulchella* collected on New Ireland, Papua New Guinea and held at the University of Kansas Biodiversity Institute and Natural History Museum (see S2 Table for details on all samples).

We collected whole blood using brachial venipuncture from birds captured in mist nets at flowering trees. We added blood to lysis buffer [90] and stored it at room temperature until arrival to the lab, where it was subsequently stored at -80°C. We extracted DNA using a Qiagen DNeasy kit with an RNase step.

Extracted genomic DNA was sequenced at Novogene (Sacramento, CA). Following quality and concentration assessment using Agarose Gel Electrophoresis and Qubit 2.0, genomic DNA was randomly fragmented and fragments were end polished, A-tailed, and ligated with Illumina adapters. Further PCR amplification preceded library construction and purification with the AMPure XP system. Finally, size distribution of libraries was checked using Agilent 2100 Bioanalyzer (Agilent Technologies, CA, USA). Libraries were then pooled and sequenced by synthesis using the Illumina platform to generate 150 bp paired end reads. The *M*. *pulchella* sample was sequenced at the Oklahoma Medical Research Foundation. Libraries were constructed using the Swift 2S Turbo DNA Library Kit prior to sequencing by synthesis of 150 bp paired end reads using the Illumina Novaseq machine.

### Reference genome assembly

We sequenced a *M*. *tristrami* female at the University of Delaware DNA sequencing & Genotyping Cener. HiFi libraries were prepared with SMRTbell prep kit, followed by Blue Pippin size selection (15-20Kbp) before sequencing on a PacBio Sequel IIe. We generated a *de novo* assembly using hifiasm v0.13-r308 with default parameters using the resulting long reads [91,92]. We used GeMoMa (v1.8) and the annotation from zebra finch genome bTaeGut1.4.pri to infer a rough annotation of genes in the *Myzomela tristrami* genome. We then used these rough annotations, comparing contigs against both zebra finch and the chicken genome bGalGal1.mat.broiler.GRCg7b to infer synteny relationships, remove duplicate haplotigs, and, finally, scaffold contigs into chromosomes in *Myzomela tristrami*. The resulting assembly uses the zebra finch numbering system for chromosomes 1–29; chromosome 30–40 were named in descending order of size. Final chromosomes and contigs were aligned with those of related species—helmeted honeyeater (*Lichenostomus melanops cassidix*), and blue-faced honeyeater (*Entomyzon cyanotis)*—using Mauve (version 2015-02-25), and visualized using FastANI (v1.33) [62,70,93,94]. We generated repetitive DNA libraries using the RepeatModeler v2 pipeline [95]. RepeatModeler employs a combination of *de novo* and homology-based characterization of different classes of repeats. The repeat library was annotated and combined with Repbase, and manually curated repeat libraries from other studies [96–99]. We then used RepeatMasker (v4.1.0) to identify and mask repetitive regions of the genome [100].

### Alignment and variant calling

We used Trim Galore (https://www.bioinformatics.babraham.ac.uk/projects/trim_galore/) to process raw reads. Trim Galore first removes low quality reads from the 3’ end and then trims adapter sequences using the program Cutadapt [101] before running FastQC (https://www.bioinformatics.babraham.ac.uk/projects/fastqc/) to check adapter content after trimming. We aligned trimmed reads to the *Myzomela tristrami* reference genome using Burrows-Wheeler-Aligner (bwa-mem, v0.7.17; [102], mapping 26,893,270,155 reads and yielding a mean coverage of 16.95x. After alignment, we sorted the resulting mapped reads by coordinate using samtools, v1.7 [103]. We continued with processing following the Genome Analysis Toolkit best practices workflow (GATK 4.2.6.1 [104]). First, we used AddOrReplaceReadGroups (Picard v.2.12.0, http://broadinstitute.github.io/picard) to denote flow cell and lane of each read. We used MarkDuplicates (Picard v12.2.0) to identify duplicate reads resulting from PCR amplification. Next, we used FixMateInformation (Picard v12.2.0) to verify and correct information between mate-pairs. At this point we assessed coverage using qualimap (v2.2.1; [105]) and confirmed sex of individuals based on coverage of Z scaffolds (diploid in males, haploid in females). In addition, we extracted mean coverage of sex-linked scaffolds and chromosomes 1–10 for allopatric *Mtris* individual to verify that mapping and raw read depth supported assignment of chr5 derived sequence in the *Mtris* reference assembly as Z- or W-linked (Fig 2).

We then called variants using the GATK pipeline, starting with HaplotypeCaller, on the diploid (default) setting. Due to hemizygosity of female sex chromosomes, we first ran HaplotypeCaller on autosomes and pseudo-autosomal regions across both sexes. We then separated males and females, running HaplotypeCaller on males for Z sequence only and on females for both Z and W sequence. We then used CombineGVCFs to facilitate joint genotyping for each region of the genome. Finally, using GenotypeGVCFs we generated an all-sites VCF file that contained both variant and invariant sites for downstream filtering and analysis.

### Filtering

We used VariantFiltration and GATK recommendations for hard filtering in non-model organisms to flag any sites that had QD < 2.0, SOR > 3.0, FS > 60.0, MQ < 40.0, MQRankSum < 12.5, or ReadPosRankSum < -80.0. We also flagged any sites overlapping known repetitive regions in the reference genome. We then used bcftools [103] to recode any flagged low-quality sites or sites in repetitive regions as missing. We used vcftools [106] to remove indels and assess depth of coverage prior to further filtering. We used bcftools to recode any autosomal genotypes as missing if they had less than 10x or more than 34x (twice the mean before filtering for depth) coverage averaged across all samples. For sex chromosome genotypes we adjusted our depth of coverage filters to accommodate the reduced coverage for hemizygous female samples, recoding as missing any genotypes less than 6x or more than 24x averaged across all samples. In addition, we further filtered sex chromosomes to remove any spurious heterozygous sites on the female Z and W sequence (excluding the new pseudo-autosomal region), recoding any such sites as missing [107]. For mitochondrial genotypes we imposed only a minimum depth filter of 10x, and also masked any regions showing heteroplasmy, resulting in 14,122 remaining sites.

For individual-level assessment of genomic variation and admixture we did further filtering, imposing a minimum minor allele frequency of 0.05 in vcftools and pruning for linkage disequilibrium (LD) in plink [108]. Our pruning procedure calculated LD between each pair of single nucleotide polymorphisms (SNPs) in 50 SNP windows, removing one SNP of each pair with an r^2^ > 0.5. The window was then shifted 5 SNPs forward before repeating the procedure.

### Population genomic analyses

To infer demographic history and effective population sizes for *M*. *cardinalis*, *M*. *tristrami* and the outgroup *M*. *pulchella*, we used individuals sampled in allopatry to construct a pairwise sequential Markovian coalescent (PSMC) model [71]. We first generated a consensus sequence in fastq format for each individual using samtools mpileup and bcftools call [103], followed by limiting to autosomes and using the vcf2fq in the vcfutil.pl of bcftools. We then ran PSMC using default settings (https://github.com/lh3/psmc), and plotted output in the R v4.1.1 [109] package ggplot2 [110], using generation length of 2.37, 2.25, and 2.51 years for *M*. *pulchella*, *M*. *cardinalis*, and *M*. *tristrami* respectively from [111], and a per generation mutation rate of 4.6*10^−9^ from [112].

For all remaining analyses we separated results for autosomes, neo-PAR, Z/neo-Z, W/neo-W, and the mitochondrial genome. For windowed estimates we further parsed ancestral sex chromosome regions from neo-sex chromosome regions. We used the program pixy [113] and an allsites VCF filtered for quality and depth to calculate nucleotide diversity (π), absolute divergence (*d*_xy_) and pairwise genetic differentiation (*F*_ST_) across 50kb windows for each population of phenotypically *cardinalis* and *tristrami* individuals. Using a quality and depth filtered VCF containing only variant sites, we used vcftools [106] to calculate Tajima’s D in 50kb windows. For all windowed analyses we limited our calculation of average estimates to 50kb windows containing a minimum of 10,000 genotyped sites, with the exception of estimates for mitochondrial sequence (which is a single window). When calculating ratios of nucleotide diversity of sex chromosomes to autosomes (S6 Table), we restricted our autosomal windows to chromosomes 1–10. Chromosomes 1–10 are similar in size to the Z/neo-Z sex chromosome, allowing more direct comparison of nucleotide diversity, as recombination rate and nucleotide diversity is expected to be elevated in smaller michrochromosomes [114]. All other estimates involving autosomes include all autosomes.

To quantify the degree of admixture across the genome we used the quality and depth filtered dataset to conduct ABBA-BABA tests in Dsuite [73,74]. We calculated *D* statistics for autosomes, neo-PAR, Z, neo-Z, and W/neo-W and used a block-jackknife procedure to determine if the *D* statistic was significantly different from zero. To quantify the proportion of introgression for each region we calculated *f*_*4*_ admixture ratio, which compares the observed excess of ABBA sites to the expected value if admixture was complete, by substituting a subset of P3 individuals for P2 individuals. The value of *f*_*4*_ therefore provides an estimate of the proportion of admixture assuming unidirectional introgression from P3 into P2 [74,115]. The assumption of unidirectional introgression may be inaccurate, so we also calculated the *f*_*dM*_ statistic, which is agnostic to the direction of gene flow. Instead, it uses the frequency of the derived allele to determine whether P2 or P3 is the donor population. The *f*_*dM*_ statistic is symmetric about zero, with positive values indicating gene flow between P2 and P3 and negative values indicating gene flow between P1 and P3. Because we have allopatric populations for both parental species, we can construct and analyze two topologies to compare amount (*f*_*dM*_) and direction (*f*_*4*_) of introgression between sympatric *Mcard* and *Mtris*. The *Mcard* P3 topology places allopatric and sympatric *Mtris* as P1 and P2 respectively and sympatric *Mcard* as P3, therefore estimating gene flow from *Mcard* into *Mtris* for *f*_*4*_. The *Mtris* P3 topology places allopatric and sympatric *Mcard* as P1 and P2 respectively, and sympatric *Mtris* as P3, giving an estimate of gene flow from *Mtris* into *Mcard* using the *f*_*4*_ admixture proportion. In addition to calculating *D*, *f*_*4*_ and *f*_*dM*_ for all autosomes combined, we also calculated these for each chromosome individually to further visualize patterns of introgression across the genome. We calculated the *f*_*dM*_ statistic in 100 SNP non-overlapping windows and averaged across windows in which the *f*_*dM*_ statistic *≥* 0, indicating potential gene flow between P2 and P3, sympatric *Mcard* and sympatric *Mtris*.

To understand genomic variation at an individual level and assess degree of admixture we conducted a principal components analysis (PCA) using the package SNPRelate [116] in R to investigate genomic variation of autosomes, Z/neo-Z, and W/neo-W among individuals sampled on Makira, Ugi and Three Sisters. For characterization of individuals as F1 or advanced generation backcross in the triangle plot we first identified 2,449 autosomal and 37,613 Z/neo-Z SNPs fixed between species (*F*_ST *=*_ 1) using allopatric individuals in vcftools. Using only the fixed SNPs we then calculated interspecific heterozygosity and hybrid index on the autosomes for all sympatric individuals and on the Z/neo-Z using only sympatric males in the package introgress in R [117]. Finally, we used ADMIXTURE [76] which estimates a maximum likelihood proportion of ancestry per individual and uses cross-validation error to determine the optimal number of populations or groups (K) present in sample of individuals. We ran replicate analyses using values of K from 1 to 7, and performed fivefold cross-validation to estimate error associated with each value of K.

We generated a mitochondrial haplotype network using variant sites from the full mitochondrial genome filtered for quality, minimum depth, and heteroplasmy. We used vcf2phylip [118] to convert the VCF to a phylip file for importing into the program PopART [119], where we constructed a TCS network [120] to visualize haplotype sharing and mutations separating all individuals in our dataset (including phenotypic hybrids and the outgroup *M*. *pulchella*).

To assess the number of private alleles for each species and sampling location we used a custom perl script (https://github.com/ehshogren/MyzomelaPopulationGenomics) which identified monomorphic or biallelic sites that were found only in the species under consideration and present in at least five individuals, reporting which location(s) the individuals carrying that private allele were sampled from. To identify the number of fixed differences and shared polymorphisms between species/sampling locations we used another custom perl script which considered two groups of phenotypically *Mcard* or *Mtris* individuals (from different species and/or sampling locations) and required a minimum of 5 individuals in each group with a genotype at the site in question.

## Supporting information

S1 Table Reference genome summary. Summary of *Myzomela tristrami* reference genome statistics, including both the raw and final scaffolded assembly.
(PDF)

S2 Table Whole genome sampling. Number of whole genome sequences by species, sampling site, and sex.
(PDF)

S3 Table SNPs per genomic region. Number of single nucleotide polymorphisms per genomic region for different filtering of datasets used in analyses.
(PDF)

S4 Table Nucleotide diversity, allopatric *cardinalis* sampling sites split. Nucleotide diversity (π) averaged across 50 kb windows, standard error in parentheses for each sampled population of phenotypic parental *Myzomela cardinalis* and *M*. *tristrami*.
(PDF)

S5 Table Nucleotide diversity ratios. Ratio of nucleotide diversity for sex chromosome regions to large autosomes (chr1–10), and for the new pseudo-autosomal region (neoPAR) to a comparatively sized autosome (chr 14). Nucleotide diversity averaged across 50kb windows.
(PDF)

S6 Table Differentiation between populations, allopatric *cardinalis* sampling sites split. Differentiation (F_ST_), averaged across 50kb windows for each region of the genome, standard error in parentheses for pairwise comparisons of phenotypic parental populations of *Myzomela cardinalis* and *M*. *tristrami*. Allopatry and sympatry abbreviated as Allo. and Sym. respectively.
(PDF)

S7 Table Divergence between populations. Divergence (*d*_*xy*_) averaged across 50kb windows for each region of the genome, standard error in parentheses for pairwise comparisons of phenotypic parental populations of *Myzomela cardinalis* and *M*. *tristrami*. Allopatry and sympatry abbreviated as Allo. and Sym. respectively.
(PDF)

S8 Table Expectations, characteristics of genomic compartments. Summary of expectations for genomic compartments with respect to recombination, ploidy, and introgression (relative to autosomes).
(PDF)

S9 Table Contingency table for Fisher’s exact test of W/neo-W and mtDNA phenotype-genotype matching. Contingency tables of number of individuals with matching or mismatched phenotype and W/neo-W or mitochondrial genotype. Number of individuals differs between W/neo-W and mitochondria because W/neo-W includes only females while mitochondria includes both females and males. Fisher’s exact test significant for W/neo-W (*p* = 0.026) and for mitochondria (*p* = 0.005).
(PDF)

S10 Table Sex ratios for molecularly sexed individuals. Number of captured and molecularly sexed males and females for each sampled population, used to calculate a sex ratio.
(PDF)

S11 Table Private alleles. Number of alleles per genomic region private to each population of the respective focal species, using SNPs filtered for depth and quality only.
(PDF)

S12 Table Fixed and shared alleles. Number of fixed and shared alleles in each region of the genome for population comparisons, using SNPs filtered for depth and quality only. Allopatry and sympatry abbreviated as Allo. and Sym. respectively.
(PDF)

S1 Fig Comparative structure of neo-sex chromosomes. Comparative structure of neo-sex chromosomes across *Lichenostomus melanops cassidix* (no neo-sex chromosome), *Myzomela tristrami* (neo-sex chromosome), and *Entomyzon cyanotis* (neo-sex chromosome, described in Burley and Orzechowski et al. 2023 [62]).
(PDF)

S2 Fig *f*_*4*_ admixture ratio, all topologies. Admixture ratio (*f*_*4*_ statistic) for each autosome and sex chromosome regions. Color of the point indicates for which topology the statistic was calculated, and shape of the point indicates whether the *D* statistic for that chromosome was significantly different from zero, using the block-jackknife procedure.
(PDF)

S3 Fig ADMIXTURE cross-validation error and plot of autosomes at K = 2. Cross-validation error for ADMIXTURE of K = 1–7 for autosomes (A), Z/neo-Z (B), and W/neo-W (C). ADMIXTURE plot for autosomal sequence with K = 2 (D). Phenotypic hybrids are outlined in yellow, with phenotypic *cardinalis* to the left and phenotypic *tristrami* to the right of phenotypic hybrids.
(PDF)

S4 Fig *f*_*dM*_ values vs. chromosome length. Metric for quantifying admixture (*f*_*dM*_) plotted against length of chromosome or chromosomal region (shape indicates genomic compartment). We calculated and averaged *f*_*dM*_ across 100 SNP non-overlapping windows using both the *cardinalis* P3 and the *tristrami* P3 topology (color of shapes indicates topology). We included only windows where *D* ≥ 0 indicating no introgression or sharing of alleles between sympatric populations (P2 and P3).
(PDF)

S5 Fig Principal component 1 of autosomes vs. sex chromosomes. Principal component 1 (PC1) of autosomal PCA plotted against PC1 of Z/neo-Z region (A) and W/neo-W region (B). Symbol color represents phenotypic species assignment while symbol shape indicates sampling locality. Plot for Z/neo-Z is separated by sex to distinguish homogametic males and heterogametic females. All individuals are female in (B).
(PDF)

S6 Fig *f*_*dM*_ values vs. allopatric *Mtris* nucleotide diversity. Metric for quantifying admixture (*f*_*dM*_) plotted against nucleotide diversity of chromosome or chromosomal region for the *Mtris* allopatric population (shape indicates genomic compartment). We calculated and averaged *f*_*dM*_ across 100 SNP non-overlapping windows using the *cardinalis* P3 topology. We included only windows where *D* ≥ 0 indicating no introgression or gene flow between sympatric *Mtris* (P2) and sympatric *Mcard* (P3). When considering only autosomes there is a significant correlation between *f*_*dM*_ and nucleotide diversity (Pearson’s correlation coefficient = 0.41, *p* = 0.007).
(PDF)

S7 Fig Mitochondrial haplotype network. Mitochondrial haplotype TCS network show that *Mcard* haplotypes are much less diverse than *Mtris* haplotypes but are shared with hybrids and phenotypic *Mtris* individuals in sympatry. Mutations between nodes shown in parentheses, and number of individuals sharing haplotype denoted by size of circles (see legend).
(PDF)
