# Supplementary material for: Recent secondary contact, genome-wide admixture, and asymmetric introgression of neo-sex chromosomes between two Pacific island bird species
Source: PLoS Genet. 2024 Aug 22;20(8):e1011360. doi: 10.1371/journal.pgen.1011360 (PMC11340901; doi:10.1371/journal.pgen.1011360)
Supplement: S1 Table — Summary of Myzomela tristrami reference genome statistics, including both the raw and final scaffolded assembly. (PDF) [file pgen.1011360.s001.pdf]

S1 Table: Reference genome summary

	Final scaffolded assembly		Raw assembly	
	**primary**	**alternate**	**primary**	**alternate**
**total length**	1257779490	1004978486	1505700183	757038143
**number of contigs**	250	1272	354	1193
**largest contig**	160265273	19080087	102193978	16102540
**N50**	41923986	5173992	25668304	4305385
**GC%**	42.91	42.74	43.23	42.04
**BUSCO** (passeriformes odb10)				
**complete**	96.60%	88%	96.60%	66.6
single-copy	92.9	87.3	71.6	66
multi-copy	3.7	0.7	25	0.6
**fragmented**	0.6	0.6	0.6	0.6
**missing**	2.8	11.4	2.8	32.8

Summary of *Myzomela tristrami* reference genome statistics, including both the raw and final scaffolded assembly. Note that the primary assembly is partially diploid, as it includes both neo-Z and neo-W contigs.
