# Supplementary material for: Recent secondary contact, genome-wide admixture, and asymmetric introgression of neo-sex chromosomes between two Pacific island bird species
Source: PLoS Genet. 2024 Aug 22;20(8):e1011360. doi: 10.1371/journal.pgen.1011360 (PMC11340901; doi:10.1371/journal.pgen.1011360)
Supplement: S2 Table — Number of whole genome sequences by species, sampling site, and sex. (PDF) [file pgen.1011360.s002.pdf]

S2 Table: Whole-genome resequenced samples

Species	Site	Male	Female
Myzomela cardinalis	Ugi	9	11
	Three Sisters	10	10
	Sympatry (Makira)	20	20
Myzomela tristrami	Allopatry	10	10
	Sympatry (Makira)	15	15
Phenotypic hybrid	Sympatry (Makira)	8	4
Myzomela pulchella	New Ireland		1

Number of whole genome sequences by species, sampling site, and sex. The *M. pulchella* sample is KU specimen catalog # 121498, KU tissue sample # 27751.
