# Supplementary material for: Recent secondary contact, genome-wide admixture, and asymmetric introgression of neo-sex chromosomes between two Pacific island bird species
Source: PLoS Genet. 2024 Aug 22;20(8):e1011360. doi: 10.1371/journal.pgen.1011360 (PMC11340901; doi:10.1371/journal.pgen.1011360)
Supplement: S3 Table — Number of single nucleotide polymorphisms per genomic region for different filtering of datasets used in analyses. (PDF) [file pgen.1011360.s003.pdf]

S3 Table: SNPs per genomic region

filtered dataset	autosome	neo-PAR	Z	neo-Z	W/neo-W	mtDNA
Quality + depth	27,517,646	601,181	1,431,349	641,775	90,926	1060
	autosome		Z/neo-Z			
Quality + depth +<br>maf > 0.05 + LD pruned	2,655,867		114,311		1,915	NA

Number of single nucleotide polymorphisms per genomic region for different filtering of datasets used in analyses.
