# Supplementary material for: Recent secondary contact, genome-wide admixture, and asymmetric introgression of neo-sex chromosomes between two Pacific island bird species
Source: PLoS Genet. 2024 Aug 22;20(8):e1011360. doi: 10.1371/journal.pgen.1011360 (PMC11340901; doi:10.1371/journal.pgen.1011360)
Supplement: S4 Table — Nucleotide diversity (π) averaged across 50 kb windows, standard error in parentheses for each sampled population of phenotypic parental Myzomela cardinalis and M. tristrami. (PDF) [file pgen.1011360.s004.pdf]

S4 Table: Nucleotide diversity (π), allopatric *cardinalis* sampling sites split

population	autosome	neo-PAR	Z	neo-Z	W	neo-W	mtDNA
Myzomela cardinalis							
Ugi	0.002398 (7×10^{-6})	0.002675 (3.9×10^{-5})	0.001076 (2×10^{-5})	0.001076 (2.4×10^{-5})	3e-06 (1×10^{-6})	3e-06 (0)	0.000233 (NA)
Three Sisters	0.00198 (7×10^{-6})	0.002135 (4.1×10^{-5})	0.000588 (1.6×10^{-5})	0.000356 (1.4×10^{-5})	6e-06 (1×10^{-6})	5e-06 (0)	0.000444 (NA)
Sympatry	0.002468 (7×10^{-6})	0.002617 (4.1×10^{-5})	0.001008 (1.9×10^{-5})	0.000711 (2.1×10^{-5})	5e-06 (1×10^{-6})	4e-06 (0)	0.000235 (NA)
Myzomela tristrami							
Allopatry	0.002759 (9×10^{-6})	0.00336 (4.2×10^{-5})	0.001738 (2×10^{-5})	0.001458 (1.7×10^{-5})	2.3e-05 (2×10^{-6})	2e-05 (0)	0.001253 (NA)
Sympatry	0.002871 (9×10^{-6})	0.003439 (4.1×10^{-5})	0.001765 (2.1×10^{-5})	0.001474 (1.7×10^{-5})	0.000587 (1.5×10^{-5})	0.000557 (4×10^{-6})	0.013763 (NA)

Nucleotide diversity (π) averaged across 50 kb windows, standard error in parentheses for each sampled population of phenotypic parental *Myzomela cardinalis* and *M. tristrami*.
