# Supplementary material for: Recent secondary contact, genome-wide admixture, and asymmetric introgression of neo-sex chromosomes between two Pacific island bird species
Source: PLoS Genet. 2024 Aug 22;20(8):e1011360. doi: 10.1371/journal.pgen.1011360 (PMC11340901; doi:10.1371/journal.pgen.1011360)
Supplement: S5 Table — Ratio of nucleotide diversity for sex chromosome regions to large autosomes (chr1–10), and for the new pseudo-autosomal region (neoPAR) to a comparatively sized autosome (chr 14). Nucleotide diversity averaged across 50kb windows. (PDF) [file pgen.1011360.s005.pdf]

S5 Table: Nucleotide diversity ratios

population	Chr1 -10 π (A)	Chr14 π	neo-PAR/A	neo-PAR/ chr14	Z/A	neo-Z/A	W/A	neo-W/A
Myzomela cardinalis								
Ugi	0.00238	0.00249	1.124	1.074	0.452	0.452	0.001	0.001
Three Sisters	0.00197	0.00204	1.084	1.047	0.298	0.181	0.003	0.003
Sympatry	0.00245	0.00263	1.068	0.995	0.411	0.290	0.002	0.002
Myzomela tristrami								
Allopatry	0.00269	0.00302	1.249	1.113	0.646	0.542	0.009	0.007
Sympatry	0.00281	0.00307	1.224	1.120	0.628	0.525	0.209	0.198

Ratio of nucleotide diversity for sex chromosome regions to large autosomes (chr1 – 10), and for the new pseudo-autosomal region (neo-PAR) to a comparably sized autosome (chr 14). Nucleotide diversity averaged across 50kb windows.
