# Supplementary material for: Recent secondary contact, genome-wide admixture, and asymmetric introgression of neo-sex chromosomes between two Pacific island bird species
Source: PLoS Genet. 2024 Aug 22;20(8):e1011360. doi: 10.1371/journal.pgen.1011360 (PMC11340901; doi:10.1371/journal.pgen.1011360)
Supplement: S7 Table — Divergence (dxy) averaged across 50kb windows for each region of the genome, standard error in parentheses for pairwise comparisons of phenotypic parental populations of Myzomela cardinalis and M. tristrami. Allopatry and sympatry abbreviated as Allo. and Sym. respectively. (PDF) [file pgen.1011360.s007.pdf]

S7 Table: Divergence (d_{xy}) between populations

population comparison	autosome	neo-PAR	Z	neo-Z	W	neo-W	mtDNA
Myzomela cardinalis							
Ugi *Mcard* vs. Three Sisters *Mcard*	0.002403 (7×10^{-6})	0.00264 (4×10^{-5})	0.00105 (2.1×10^{-5})	0.000901 (2.4×10^{-5})	5e-06 (1×10^{-6})	5e-06 (0)	0.000407 (NA)
Ugi *Mcard* vs. Sym. *Mcard*	0.00253 (7×10^{-6})	0.002744 (4×10^{-5})	0.001138 (2×10^{-5})	0.001014 (2.4×10^{-5})	4e-06 (1×10^{-6})	4e-06 (0)	0.000246 (NA)
Three Sisters *Mcard* vs. Sym. *Mcard*	0.002401 (7×10^{-6})	0.002574 (4.1×10^{-5})	0.000959 (2×10^{-5})	0.000704 (2.6×10^{-5})	6e-06 (1×10^{-6})	4e-06 (0)	0.000371 (NA)
Myzomela tristrami							
Allo. *Mtris* vs. Sym. *Mtris*	0.002833 (9×10^{-6})	0.003416 (4.1×10^{-5})	0.001782 (2.1×10^{-5})	0.001492 (1.7×10^{-5})	0.000396 (1×10^{-5})	0.000376 (2×10^{-6})	0.009029 (NA)
Heterospecific							
Ugi *Mcard* vs. Allo. *Mtris*	0.003421 (9×10^{-6})	0.003943 (4.2×10^{-5})	0.00307 (2.2×10^{-5})	0.003088 (2.7×10^{-5})	0.001416 (3.6×10^{-5})	0.001351 (9×10^{-6})	0.040159 (NA)
Three Sisters *Mcard* vs. Allo. *Mtris*	0.003564 (9×10^{-6})	0.004074 (4.1×10^{-5})	0.003275 (2.2×10^{-5})	0.003403 (2.7×10^{-5})	0.001419 (3.7×10^{-5})	0.001352 (9×10^{-6})	0.040208 (NA)
Allo. *Mtris* vs. Sym. *Mcard*	0.003403 (9×10^{-6})	0.003947 (4.1×10^{-5})	0.003132 (2.2×10^{-5})	0.003185 (2.9×10^{-5})	0.001432 (3.8×10^{-5})	0.001356 (9×10^{-6})	0.040205 (NA)
Ugi *Mcard* vs. Sym. *Mtris*	0.003334 (8×10^{-6})	0.003859 (4×10^{-5})	0.003054 (2.2×10^{-5})	0.003083 (2.7×10^{-5})	0.00104 (2.7×10^{-5})	0.000993 (6×10^{-6})	0.032095 (NA)
Three Sisters *Mcard* vs. Sym. *Mtris*	0.003453 (8×10^{-6})	0.003972 (3.9×10^{-5})	0.003256 (2.1×10^{-5})	0.003396 (2.7×10^{-5})	0.001043 (2.7×10^{-5})	0.000993 (6×10^{-6})	0.032163 (NA)
Sym. *Mcard* vs. Sym. *Mtris*	0.003313 (8×10^{-6})	0.003854 (3.9×10^{-5})	0.003117 (2.1×10^{-5})	0.003176 (2.8×10^{-5})	0.001052 (2.8×10^{-5})	0.000996 (6×10^{-6})	0.032131 (NA)

Divergence (d_{xy}) averaged across 50kb windows for each region of the genome, for pairwise comparisons of phenotypic parental populations of *Myzomela cardinalis* and *M. tristrami*. Allopatry and sympatry abbreviated as Allo. and Sym., respectively, standard errors in parentheses.
