# Supplementary material for: Recent secondary contact, genome-wide admixture, and asymmetric introgression of neo-sex chromosomes between two Pacific island bird species
Source: PLoS Genet. 2024 Aug 22;20(8):e1011360. doi: 10.1371/journal.pgen.1011360 (PMC11340901; doi:10.1371/journal.pgen.1011360)
Supplement: S8 Table — Summary of expectations for genomic compartments with respect to recombination, ploidy, and introgression (relative to autosomes). (PDF) [file pgen.1011360.s008.pdf]

S8 Table: Expectations, characteristics of genomic compartments

Genomic compartment	Recombination	Ploidy	Introgression relative to autosomes
**Autosomes**	Males and females	2n	…
**Neo-PAR**	Males and females	2n	≈
**Z/neo-Z**	Males only	2n in males<br>1n in females	<
**W/neo-W**	None	1n in females	<
**mtDNA**	None	1n	<

Summary of expectations for genomic compartments with respect to recombination, ploidy, and introgression (relative to autosomes).
