# Supplementary material for: Recent secondary contact, genome-wide admixture, and asymmetric introgression of neo-sex chromosomes between two Pacific island bird species
Source: PLoS Genet. 2024 Aug 22;20(8):e1011360. doi: 10.1371/journal.pgen.1011360 (PMC11340901; doi:10.1371/journal.pgen.1011360)
Supplement: S9 Table — Contingency tables of number of individuals with matching or mismatched phenotype and W/neo-W or mitochondrial genotype. Number of individuals differs between W/neo-W and mitochondria because W/neo-W includes only females while mitochondria includes both females and males. Fisher’s exact test significant for W/neo-W (p = 0.026) and for mitochondria (p = 0.005). (PDF) [file pgen.1011360.s009.pdf]

S9 Table: Contingency table for Fisher's exact test of W/neo-W and mtDNA phenotype-genotype matching

**Phenotype**	**Matching Genotype**	**Mismatched Genotype**
	W/neo-W	
Mcard	20	0
Mtris	11	4
	Mitochondria	
Mcard	40	0
Mtris	24	6

Contingency tables of number of individuals with matching or mismatched phenotype and W/neo-W or mitochondrial genotype. Number of individuals differs between W/neo-W and mitochondria because W/neo-W includes only females while mitochondria includes both females and males. Fisher's exact test significant for W/neo-W (p = 0.026) and for mitochondria (p = 0.005).
