# Supplementary material for: Recent secondary contact, genome-wide admixture, and asymmetric introgression of neo-sex chromosomes between two Pacific island bird species
Source: PLoS Genet. 2024 Aug 22;20(8):e1011360. doi: 10.1371/journal.pgen.1011360 (PMC11340901; doi:10.1371/journal.pgen.1011360)
Supplement: S10 Table — Number of captured and molecularly sexed males and females for each sampled population, used to calculate a sex ratio. (PDF) [file pgen.1011360.s010.pdf]

S10 Table: Sex ratios for molecularly sexed individuals

Population	No. Males	No. Females	Ratio M : F
M. cardinalis			
Ugi	22	12	1.8 : 1
Three Sisters	10	11	0.9 : 1
Sympatry	43	38	1.1 : 1
M. tristrami			
Allopatry	21	10	2.1 : 1
Sympatry	38	18	2.1 : 1

Number of phenotypic *Mcard* and *Mtris* captured foraging at flowering trees over course of fieldwork and subsequently molecularly sexed.
