# Supplementary material for: Recent secondary contact, genome-wide admixture, and asymmetric introgression of neo-sex chromosomes between two Pacific island bird species
Source: PLoS Genet. 2024 Aug 22;20(8):e1011360. doi: 10.1371/journal.pgen.1011360 (PMC11340901; doi:10.1371/journal.pgen.1011360)
Supplement: S11 Table — Number of alleles per genomic region private to each population of the respective focal species, using SNPs filtered for depth and quality only. (PDF) [file pgen.1011360.s011.pdf]

S11 Table: Private alleles

population	autosome	neo-PAR	Z	neo-Z	W	neo-W	mtDNA
Myzomela cardinalis							
Ugi	214660	3809	12322	4861	49	363	17
Three Sisters	92008	1769	4839	1776	34	223	5
Sympatry	45480	701	2255	554	3	0	1
Myzomela tristrami							
Allopatry	2406437	54121	143647	59560	190	1430	44
Sympatry	425142	11030	30210	11704	36	265	4

Number of alleles per genomic region private to each population of the respective focal species, using SNPs filtered for depth and quality only.
