# Supplementary material for: Recent secondary contact, genome-wide admixture, and asymmetric introgression of neo-sex chromosomes between two Pacific island bird species
Source: PLoS Genet. 2024 Aug 22;20(8):e1011360. doi: 10.1371/journal.pgen.1011360 (PMC11340901; doi:10.1371/journal.pgen.1011360)
Supplement: S12 Table — Number of fixed and shared alleles in each region of the genome for population comparisons, using SNPs filtered for depth and quality only. Allopatry and sympatry abbreviated as Allo. and Sym. respectively. (PDF) [file pgen.1011360.s012.pdf]

S12 Table: Fixed and shared alleles

population comparison	fix/share	autosome	neo-PAR	Z	neo-Z	W	neo-W	mtDNA
Myzomela cardinalis								
Ugi vs. Three Sisters	fixed	1	0	1	4	0	0	0
	shared	5267344	116120	146136	57381	14	111	2
Ugi vs. Sym. *cardinalis*	fixed	1	0	0	0	0	0	0
	shared	6486981	143167	230689	121498	27	204	4
Three Sisters vs. Sym. *cardinalis*	fixed	1	0	8	0	0	0	0
	shared	5478982	118124	147201	56183	49	365	14
Myzomela tristrami								
Allo. *tristrami* vs. Sym. *tristrami*	fixed	0	0	0	0	0	0	0
	shared	10683557	252252	477570	203873	149	1406	49
Heterospecific								
Ugi vs. Allo. *tristrami*	fixed	3096	0	24421	9320	5059	46435	519
	shared	4875384	115471	115491	51977	0	3	0
Three Sisters vs. Allo. *tristrami*	fixed	30293	523	58206	43858	5062	46315	518
	shared	3770388	88987	57178	14346	0	2	1
Ugi vs. Sym. *tristrami*	fixed	5	0	22139	8867	0	0	0
	shared	6239777	139770	136498	58864	28	134	6
Three Sisters vs. Sym. *tristrami*	fixed	29	0	51495	39846	0	0	0
	shared	5011776	109917	70380	16419	28	209	9
Allo. *tristrami* vs. Sym. *cardinalis*	fixed	13	1	131	54	5066	46360	516
	shared	6471493	137238	158534	63243	0	4	2
Sym. *cardinalis* vs. Sym. *tristrami*	fixed	0	0	106	36	1	0	0
	shared	8036690	163668	184641	71334	50	340	13

Number of fixed and shared alleles in each region of the genome for population comparisons, using SNPs filtered for depth and quality only. Allopatry and sympatry abbreviated as Allo. and Sym. respectively.
