# Supplementary material for: Recent secondary contact, genome-wide admixture, and asymmetric introgression of neo-sex chromosomes between two Pacific island bird species
Source: PLoS Genet. 2024 Aug 22;20(8):e1011360. doi: 10.1371/journal.pgen.1011360 (PMC11340901; doi:10.1371/journal.pgen.1011360)
Supplement: S1 Fig — Comparative structure of neo-sex chromosomes across Lichenostomus melanops cassidix (no neo-sex chromosome), Myzomela tristrami (neo-sex chromosome), and Entomyzon cyanotis (neo-sex chromosome, described in Burley and Orzechowski et al. 2023 [62]). (PDF) [file pgen.1011360.s013.pdf]

S1 Fig: Comparative structure of neo-sex chromosomes

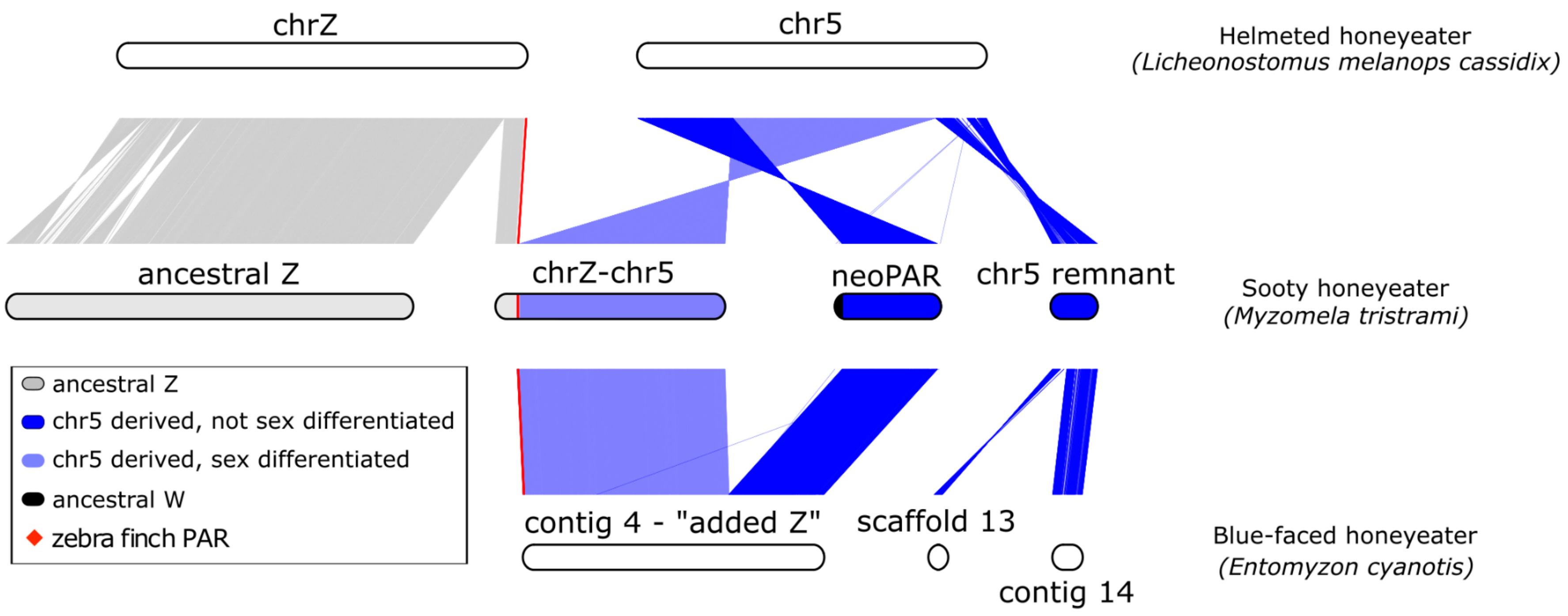

S1 Fig. Comparative structure of neo-sex chromosomes across *Lichenostomus melanops cassidix* (no neo-sex chromosome), *Myzomela tristrami* (neo-sex chromosome), and *Entomyzon cyanotis* (neo-sex chromosome, described in Burley and Orzechowski et al. 2023).
