# Supplementary material for: Recent secondary contact, genome-wide admixture, and asymmetric introgression of neo-sex chromosomes between two Pacific island bird species
Source: PLoS Genet. 2024 Aug 22;20(8):e1011360. doi: 10.1371/journal.pgen.1011360 (PMC11340901; doi:10.1371/journal.pgen.1011360)
Supplement: S2 Fig — Admixture ratio (f4 statistic) for each autosome and sex chromosome regions. Color of the point indicates for which topology the statistic was calculated, and shape of the point indicates whether the D statistic for that chromosome was significantly different from zero, using the block-jackknife procedure. (PDF) [file pgen.1011360.s014.pdf]

S2 Fig: f_4 admixture ratio, all topologies

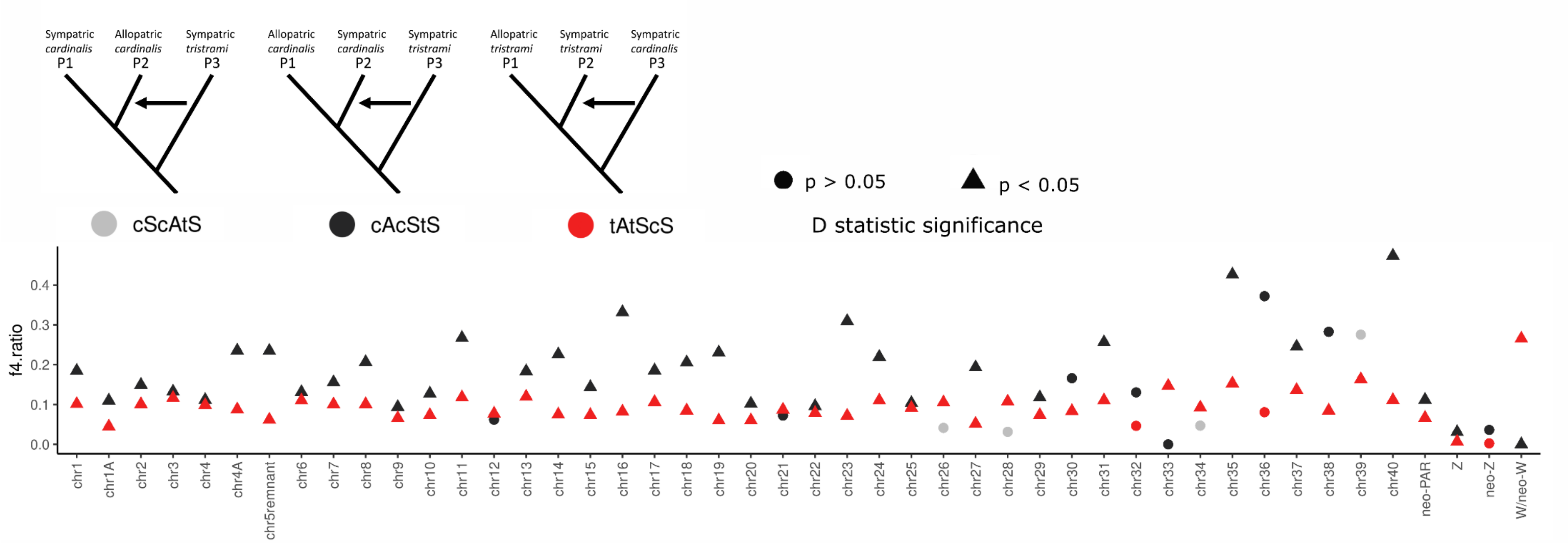

S2 Fig. Admixture ratio (*f_4* statistic) for each autosome and sex chromosome region. Color of the point indicates for which topology the statistic was calculated, and shape of the point indicates whether the *D* statistic for that chromosome was significantly different from zero, using the block-jackknife procedure.
