# Supplementary material for: Recent secondary contact, genome-wide admixture, and asymmetric introgression of neo-sex chromosomes between two Pacific island bird species
Source: PLoS Genet. 2024 Aug 22;20(8):e1011360. doi: 10.1371/journal.pgen.1011360 (PMC11340901; doi:10.1371/journal.pgen.1011360)
Supplement: S3 Fig — Cross-validation error for ADMIXTURE of K = 1–7 for autosomes (A), Z/neo-Z (B), and W/neo-W (C). ADMIXTURE plot for autosomal sequence with K = 2 (D). Phenotypic hybrids are outlined in yellow, with phenotypic cardinalis to the left and phenotypic tristrami to the right of phenotypic hybrids. (PDF) [file pgen.1011360.s015.pdf]

S3 Fig: ADMIXTURE cross-validation and plot of autosomes at K=2

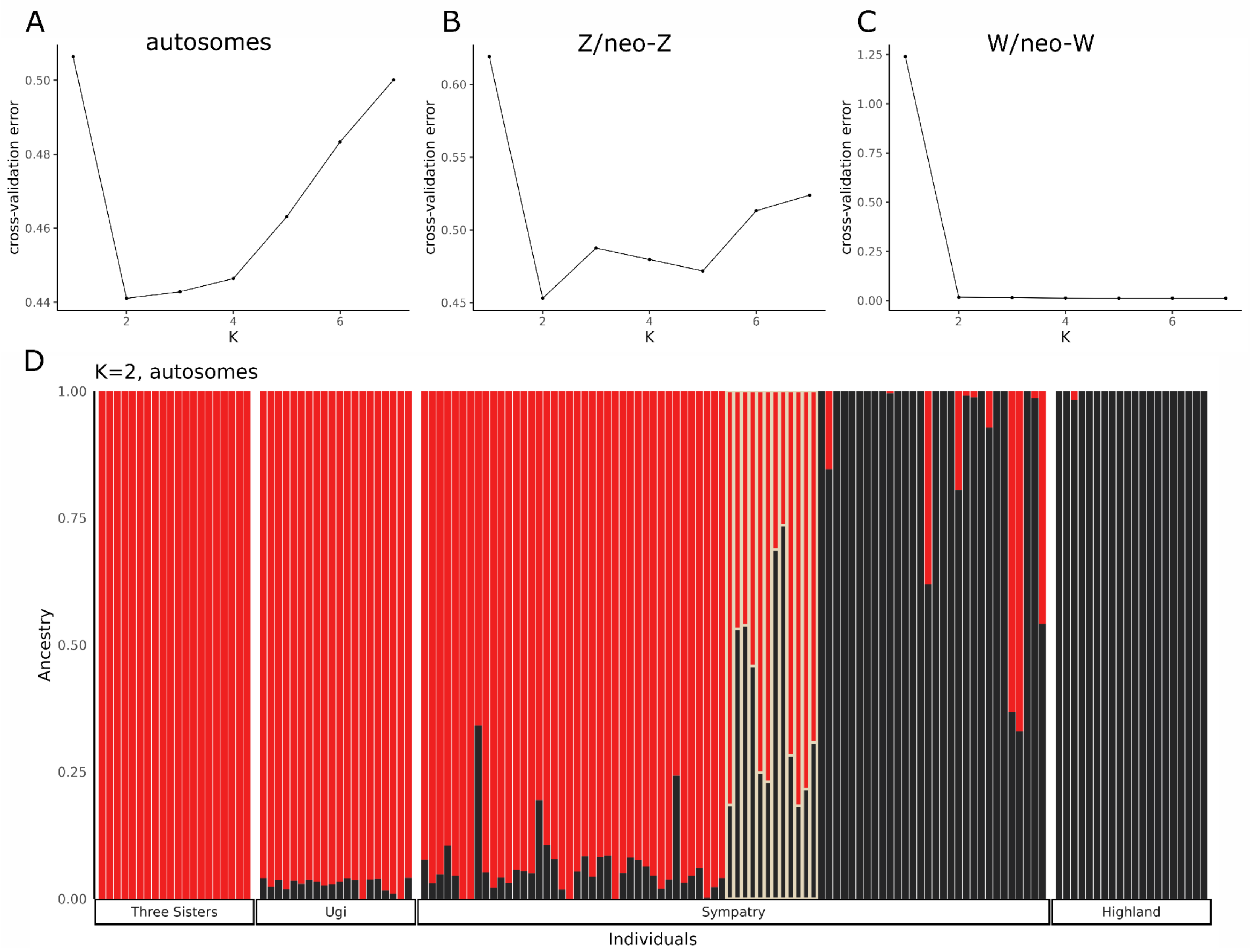

S3 Fig. Cross-validation error for ADMIXTURE of K = 1-7 for autosomes (A), Z/neo-Z (B), and W/neo-W (C). ADMIXTURE plot for autosomal sequence with K = 2 (D).
