# Supplementary material for: Recent secondary contact, genome-wide admixture, and asymmetric introgression of neo-sex chromosomes between two Pacific island bird species
Source: PLoS Genet. 2024 Aug 22;20(8):e1011360. doi: 10.1371/journal.pgen.1011360 (PMC11340901; doi:10.1371/journal.pgen.1011360)
Supplement: S4 Fig — Metric for quantifying admixture (fdM) plotted against length of chromosome or chromosomal region (shape indicates genomic compartment). We calculated and averaged fdM across 100 SNP non-overlapping windows using both the cardinalis P3 and the tristrami P3 topology (color of shapes indicates topology). We included only windows where D ≥ 0 indicating no introgression or sharing of alleles between sympatric populations (P2 and P3). (PDF) [file pgen.1011360.s016.pdf]

S4 Fig: f_{dM} vs. chromosome length

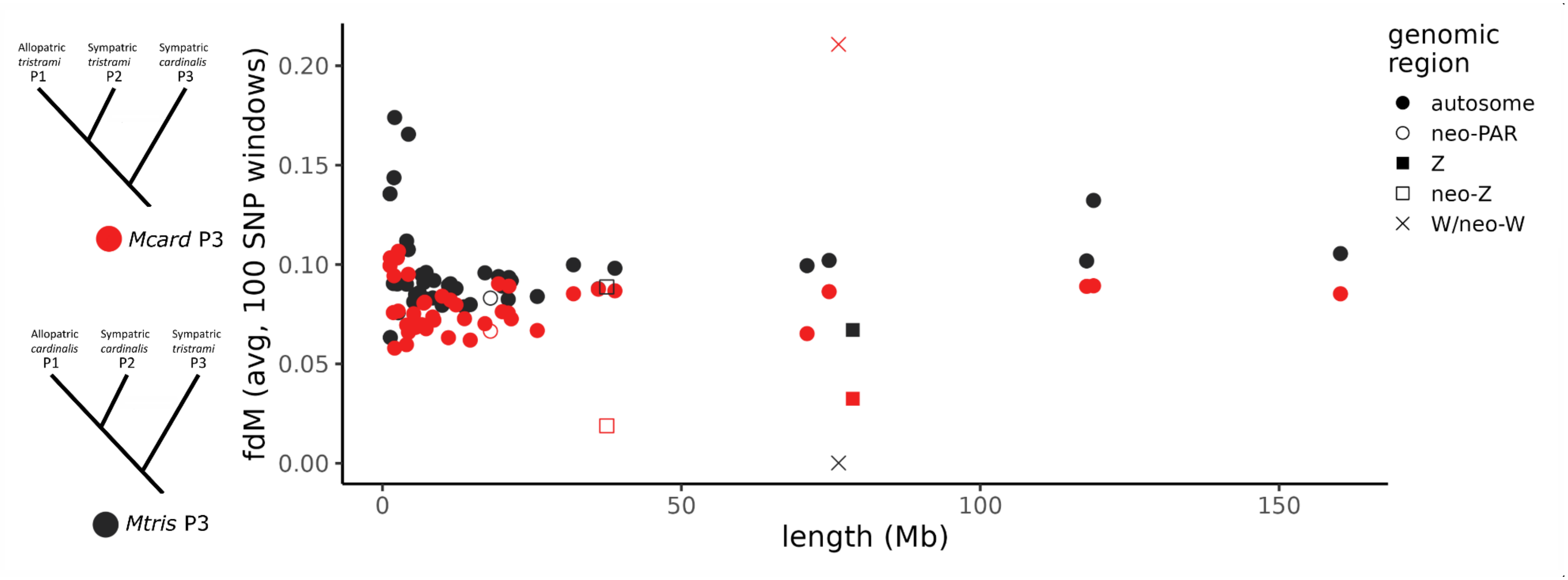

S4 Fig. Admixture statistic f_{dM} calculated in 100 SNP non-overlapping windows and averaged across the chromosome, plotted against map length for relevant chromosome or region. Different genomic regions shown in shape of point.
