# Supplementary material for: Recent secondary contact, genome-wide admixture, and asymmetric introgression of neo-sex chromosomes between two Pacific island bird species
Source: PLoS Genet. 2024 Aug 22;20(8):e1011360. doi: 10.1371/journal.pgen.1011360 (PMC11340901; doi:10.1371/journal.pgen.1011360)
Supplement: S5 Fig — Principal component 1 (PC1) of autosomal PCA plotted against PC1 of Z/neo-Z region (A) and W/neo-W region (B). Symbol color represents phenotypic species assignment while symbol shape indicates sampling locality. Plot for Z/neo-Z is separated by sex to distinguish homogametic males and heterogametic females. All individuals are female in (B). (PDF) [file pgen.1011360.s017.pdf]

S5 Fig: Principal component 1 of autosomes vs. sex chromosomes

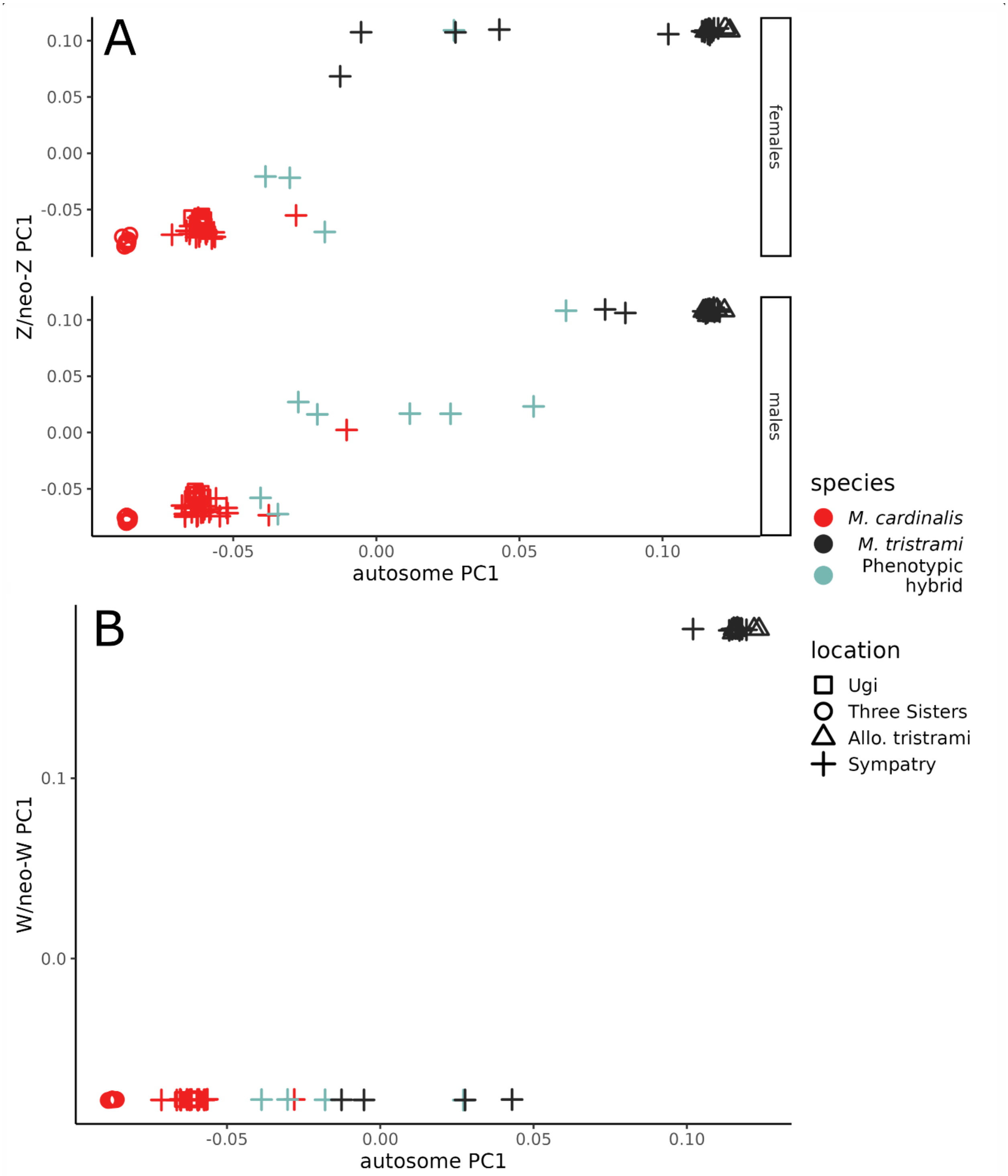

S5 Fig. Principal component 1 (PC1) of autosomal PCA plotted against PC1 of Z/neo-Z region (A) and W/neo-W region (B). Symbol color represents phenotypic species assignment while symbol shape indicates sampling locality. Plot for Z/neo-Z is separated by sex to distinguish homogametic males and heterogametic females. All individuals are female in (B).
