# Supplementary material for: Recent secondary contact, genome-wide admixture, and asymmetric introgression of neo-sex chromosomes between two Pacific island bird species
Source: PLoS Genet. 2024 Aug 22;20(8):e1011360. doi: 10.1371/journal.pgen.1011360 (PMC11340901; doi:10.1371/journal.pgen.1011360)
Supplement: S6 Fig — Metric for quantifying admixture (fdM) plotted against nucleotide diversity of chromosome or chromosomal region for the Mtris allopatric population (shape indicates genomic compartment). We calculated and averaged fdM across 100 SNP non-overlapping windows using the cardinalis P3 topology. We included only windows where D ≥ 0 indicating no introgression or gene flow between sympatric Mtris (P2) and sympatric Mcard (P3). When considering only autosomes there is a significant correlation between fdM and nucleotide diversity (Pearson’s correlation coefficient = 0.41, p = 0.007). (PDF) [file pgen.1011360.s018.pdf]

S6 Fig: f_{dM} vs. allopatric *Mtris* nucleotide diversity

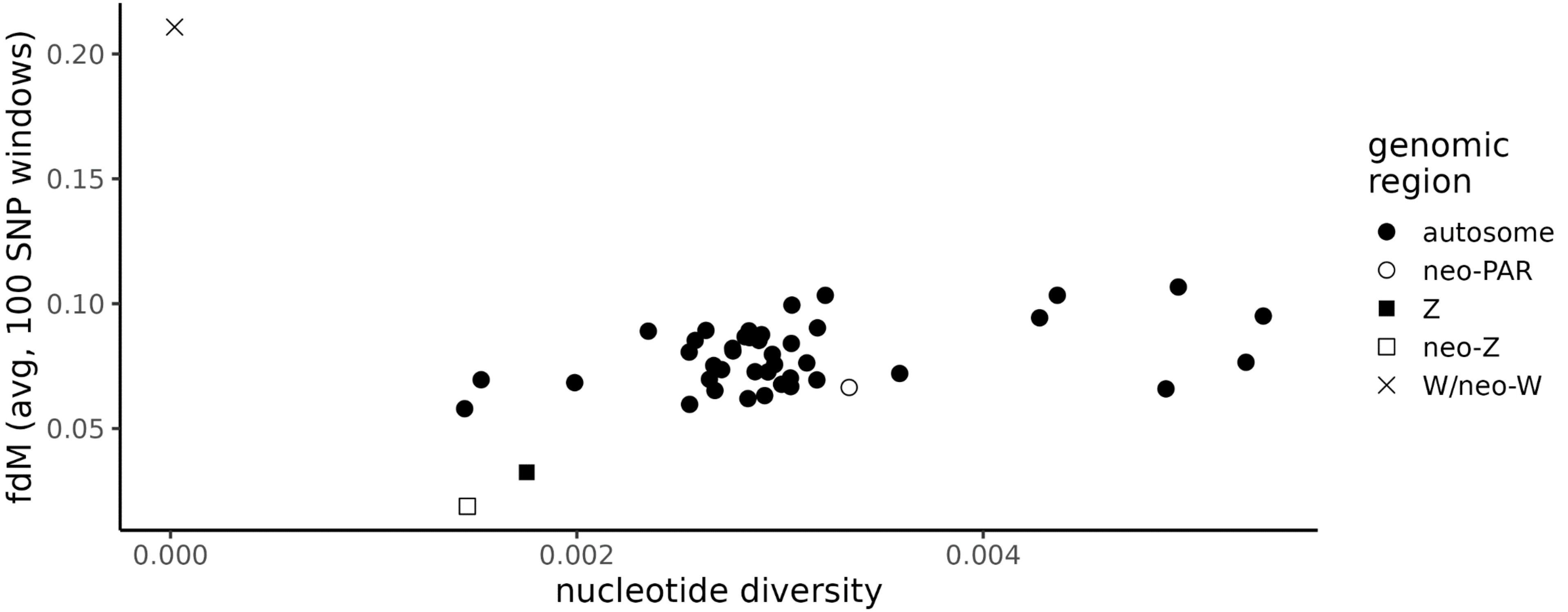

S6 Fig. Metric for quantifying admixture (f_{dM}) plotted against nucleotide diversity of chromosome or chromosomal region for the *Mtris* allopatric population (shape indicates genomic compartment). We calculated and averaged f_{dM} across 100 SNP non-overlapping windows using the *cardinalis* P3 topology. We included only windows where $D \geq 0$ indicating introgression or gene flow between sympatric *Mtris* (P2) and sympatric *Mcard* (P3). When considering only autosomes there is a significant correlation between f_{dM} and nucleotide diversity (Pearson's correlation coefficient = 0.41, p = 0.007).
