# Supplementary material for: Recent secondary contact, genome-wide admixture, and asymmetric introgression of neo-sex chromosomes between two Pacific island bird species
Source: PLoS Genet. 2024 Aug 22;20(8):e1011360. doi: 10.1371/journal.pgen.1011360 (PMC11340901; doi:10.1371/journal.pgen.1011360)
Supplement: S7 Fig — Mitochondrial haplotype TCS network show that Mcard haplotypes are much less diverse than Mtris haplotypes but are shared with hybrids and phenotypic Mtris individuals in sympatry. Mutations between nodes shown in parentheses, and number of individuals sharing haplotype denoted by size of circles (see legend). (PDF) [file pgen.1011360.s019.pdf]

S7 Fig: Mitochondrial haplotype network

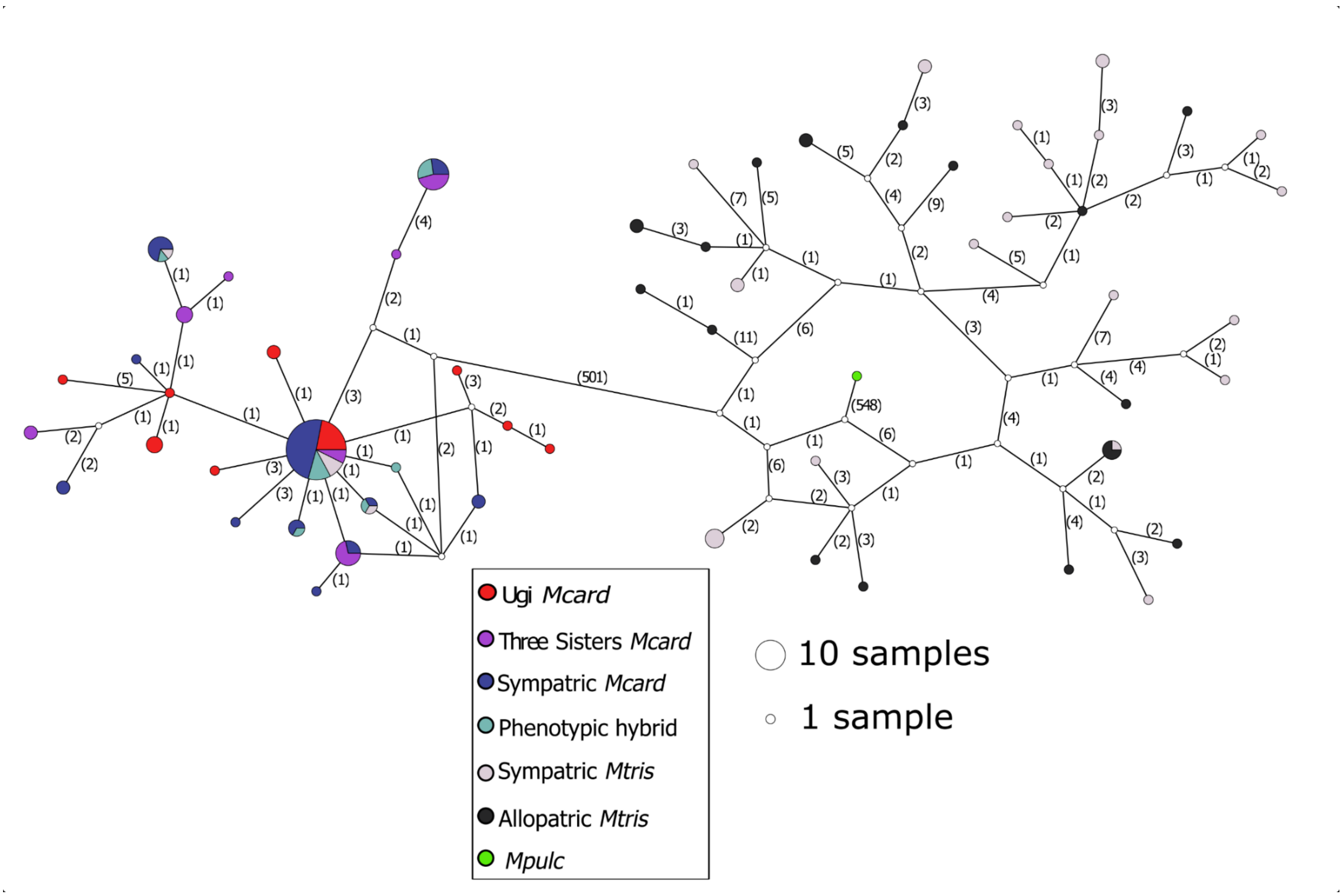

S7 Fig. Mitochondrial haplotype TCS network show that *Mcard* haplotypes are much less diverse than *Mtris* haplotypes but are shared with hybrids and phenotypic *Mtris* individuals in sympatry. Mutations between nodes shown in parentheses, and number of individuals sharing haplotype denoted by size of circles (see legend).
